# Gene expression and machine learning techniques uncover corneal biomarkers associated with oxidative stress in the myopia progression

**DOI:** 10.1038/s41598-026-46896-x

**Published:** 2026-03-30

**Authors:** Qingyi Zhou, Mingxia Ye, Zhiyong Zhang, Hongbo Luo, Yonggang Zhang, Hailan Zhao

**Affiliations:** 1 Department of Ophthalmology, Zhejiang Provincial People’s Hospital (Affiliated People’s Hospital), Hangzhou Medical College, Hangzhou, Zhejiang China; 2 Hangzhou MSK Eye Hospital, Hangzhou, Zhejiang China

**Keywords:** Myopia, Oxidative stress, Cornea, Machine learning, Regulatory network, Biomarkers, Computational biology and bioinformatics, Disease prevention, Refractive errors

## Abstract

This study aimed to identify and validate corneal biomarkers underlying myopia using human corneal tissues. Gene expression data were analyzed to investigate myopia development, where candidate genes were selected by identifying differentially expressed genes and intersecting them with oxidative stress-related genes. Machine learning techniques were employed to identify key biomarkers, and a nomogram was constructed to predict myopia risk. Utilizing corneal stromal tissues from patients who undergoing Small Incision Lenticule Extraction (SMILE) surgery, the expression levels of ATF3, GRIN2B, and GSTM3 were found to be significantly lower in the high myopia group (≤ -6.00 D) compared to the low myopia group (≥ -3.00 D and < 0 D). These biomarkers were also found to be closely associated with differential immune cell infiltration, particularly involving CD8 + T cells and eosinophils. A diagnostic nomogram was developed and showed strong discriminative potential in the discovery set. However, its predictive performance and clinical utility should be further validated in independent cohorts. ATF3, GRIN2B, and GSTM3 have emerged as promising oxidative stress-related biomarkers with significant potential for understanding myopia pathology.

**Supplementary Information:**

The online version contains supplementary material available at 10.1038/s41598-026-46896-x.

## Introduction

Myopia, a common refractive error, is characterized by blurred when viewing distant objects^1^. The global prevalence of myopia has been increasing steadily in recent year, making it a significant public health concern^2^. Several risk factors for myopia progression are commonly observed in daily life, including prolonged near work (e.g., reading and screen use) and limited time spent outdoors with reduced exposure to natural light^3^. High myopia, defined as a severe degree of nearsightedness, can lead to irreversible ocular complications such as retinal detachment, myopic maculopathy, and glaucoma^4^. In the most severe cases, these complications may result in permanent visual impairment^5^. Despite its growing prevalence and potentially devastating consequences, the etiopathogenesis of myopia progression is not fully understood. Therefore, further investigation into the etiopathogenesis of myopia is urgently needed to identify potential therapeutic targets and develop evidence-based strategies for clinical management.

Oxidative stress refers to a state of imbalance between the production of reactive oxygen species (such as superoxide anion, hydroxyl radicals, hydrogen peroxide, etc.) and the antioxidant defense system in the body under the stimulation of internal and external environmental factors^6^. Numerous studies have demonstrated a close association between oxidative stress and the onset and progression of myopia. For instance, Qi Yu^7^ and Salvador Mérida^8^ demonstrated that during the development of axial myopia, oxidative stress responses are present in the aqueous humor of the eye, and their intensity positively correlates with the elongation of the ocular axis. Furthermore, Feng-Juan Yu^9^ discovered that oxidative stress occurring in the retina, following the induction of myopia in chickens through lens application, may be implicated in the development of myopia. Despite these insights, the precise mechanisms by which oxidative stress influences myopia development remain unclear.

The cornea, occupying the anterior one-sixth of the eyeball’s outer layer, accounts for approximately two-thirds of the eye’s total refractive power. Studies have demonstrated that oxidative stress can directly compromise the barrier function established by corneal endothelial cells, leading to alterations in corneal tissue hydration that subsequently affect corneal transparency and refractive power^10^. Multiple animal studies have confirmed that modifying ocular refractive power through lens application can successfully induce myopia^11–13^. This raises a crucial question: could oxidative stress-induced changes in corneal refractive power also contribute to myopia development? The cornea’s advantageous anatomical position offers considerable convenience for clinical diagnosis and pharmacological intervention. Nevertheless, current research on the pathogenesis of myopia has paid relatively limited attention to corneal mechanisms.

This study integrated gene expression datasets (GSE112155, GSE151631, GSE136701) to identify myopia-associated differential genes, which were intersected with oxidative stress-related genes (OSRGs) to define candidates. Biomarkers were subsequently screened via machine learning and used to construct a diagnostic nomogram. Their causal relationship with myopia was further supported by Mendelian randomization (MR) analysis. Functional mechanisms were elucidated through immune infiltration profiling, Gene Ontology (GO) and Kyoto Encyclopedia of Genes and Genomes (KEGG), and regulatory network prediction. Finally, drug prediction coupled with molecular docking identified potential therapeutics, with findings preliminarily validated in human corneal tissues from myopic patients.

## Materials and methods

### Data source

The myopic datasets GSE112155 and GSE151631 were from the GEO database (https://www.ncbi.nlm.nih.gov/geo/), with sequencing platforms GPL18573 and GPL16791, respectively. GSE112155 included 20 corneal tissue samples (keratoconus: myopic corneas = 10:10). GSE151631 included 26 corneal tissue samples, comprising 19 keratoconus and 7 normal controls. After eliminating batch effects with the ComBat function from the sva package (v 3.46.0), myopic samples from GSE112155 and normal samples from GSE151631 were selected to form a dataset consisting of 10 myopic and 7 normal control samples^14^. Another dataset, GSE136701, also from the GEO database on platform GPL570, included six lens epithelial cell tissue samples—three from highly myopic individuals and three from individuals with normal vision. OSRGs were downloaded from the GeneCards database (https://www.genecards.org/), totaling 631 genes.

### Differential expression analysis and identification of candidate genes

For the count data of merge dataset and GSE136701, DEGs were generated from samples in myopic and control groups using limma package (v 3.54.2)^15^, and the filtering conditions were *P <* 0.05 and |log_2_FC| > 0.5. It was worth noting that the top 5 DEGs in terms of upward/downward p-values were labeled in the volcano and heat maps. After identifying DEGs, the intersection of DEGs and OSRGs was determined resulting in a collection of candidate genes. Subsequently, we employed the clusterProfiler (v 4.6.2)^16^ and enrichplot packages (v 1.18.4) to conduct GO and KEGG enrichment analyses (*P <* 0.05)^17–20^. This was done to explore common functions and related biological pathways among these genes.

### Identification of biomarkers and nomogram creation

To further screen for biomarkers, we applied two machine learning approaches, Least Absolute Shrinkage and Selection Operator (LASSO) and Boruta, to select candidate genes. The LASSO regression analysis was implemented using the glmnet package (v 4.1–8.1)^21^. To ensure model generalizability and mitigate overfitting, a three-fold cross-validation was employed all samples (owing to the relatively limited sample size) to determine the optimal penalty parameter (λ). The value of λ that yielded the minimum mean cross-validated error (λ = 0.0006051017) was selected to identify the most predictive feature genes. Simultaneously, the Boruta algorithm, executed via the Boruta package (v 8.0.0)^22^, was applied as a robust all-relevant feature selection method. This analysis was run with ntree set to 1000 to ensure feature importance estimation stability, while other parameters were kept at their default settings. Finally, the ggvenn package was used to identify the intersecting genes from the outputs of both the LASSO and Boruta approaches, which were defined as candidate biomarkers. To address model interpretability and assess the biological relevance of the machine learning predictions, we performed a SHapley Additive exPlanations (SHAP) analysis using the shapviz package (v 0.10.2) based on a XGBoost model. The model was configured with max_depth = 10, eta = 0.1, and lambda = 0.1, and was trained using 5-fold cross-validation. Finally, we analyzed the expression levels of candidate biomarkers across both the merge dataset and an independent validation cohort (GSE136701) to enhance the reliability of biomarker selection. Only candidate biomarkers showing significant differences and consistent patterns were selected as the final biomarkers.

A nomogram was established via the rms package (v 6.8.1) in the merge dataset to estimate the probability of myopic occurrence by incorporating these biomarkers. To evaluate the predictive accuracy of the nomogram, a calibration curve was constructed. Moreover, receiver operating characteristic (ROC) curves were used to assess the nomogram’s diagnostic performance and to compare the efficacy of each individual biomarker. Finally, we assess the clinical utility of the diagnostic model by decision curve analysis (DCA).

### Immune infiltration analysis and chromosomal localization analysis

The relationship between disease and immunity was investigated using the CIBERSORT algorithm and LM22 gene set^23^ to calculate the proportions of 22 immune cell types in disease and control samples. The infiltration abundance percentages of these immune cells were visualized using the ggplot2 package (v 3.5.1) in bar chart format. Subsequently, Wilcoxon rank-sum tests were conducted, and the proportions of the 22 immune cell types were illustrated using box plots. To delve deeper into the connection between immune cells and biomarkers, all immune cell types were selected for Spearman correlation analysis with biomarkers, and a correlation heatmap was created using the ggplot package. In addition, we use the RCircos package (v 1.2.2) for chromosomal position analysis of biomarkers.

### MR analysis

To delve deeper into the causal relationship between biomarkers and myopia, this study employed MR analysis, considering biomarkers as the exposure and myopia as the outcome. The MR analyses were based on three fundamental assumptions: (1) there is a significant correlation between the instrumental variables (IVs) and the biomarkers; (2) IVs are not associated with confounders; and (3) IVs influence outcomes solely through the biomarkers and not via other pathways.

Initially, cis-eQTL data for biomarkers were obtained from the eQTLGen database (*P <* 5 × 10^− 8^), and linkage disequilibrium filtering of SNPs was conducted using the ieugwasr package (r^2^ = 0.1 and kb = 100). Myopia data (finngen_R11_H7_MYOPIA) was downloaded from the FREEZE 11 database (https://r11.finngen.fi/). The F-statistics for each IVs were calculated to exclude weak instruments with F-values less than 10. Concurrently, confounders were excluded using the GWAS Catalog database, setting a threshold of *P <* 1 × 10^− 5^ to filter out SNPs potentially related to the outcome GWAS traits.

During the MR analysis phase, effect alleles and magnitudes were harmonized using the harmonise_data function in the TwoSampleMR package (v 0.6.3), excluding IVs significantly associated with the outcome. MR analysis was then conducted combining five methods (MR Egger, Weighted median, Inverse variance weighted (IVW), Simple mode, and Weighted mode) using the mr function.

To ensure the reliability of the MR results, we conducted several sensitivity analyses. Initially, heterogeneity was evaluated with Cochran’s Q test (*P* > 0.05). Subsequently, a horizontal pleiotropy test was performed to identify potential confounders using the mr_pleiotropy_test and MR-PRESSO functions (*P* > 0.05). To evaluate the stability of the results, we conducted leave-one-out (LOO) sensitivity analyses by sequentially excluding each SNP.

### Enrichment analysis of biomarkers

To delve deeper into the biological functions associated with biomarkers, Spearman correlation analyses were conducted between each biomarker and all genes. Following this, Gene Set Enrichment Analysis (GSEA) were executed using the clusterProfiler package. The background gene sets, c2.cp.kegg_legacy.v2024.1.Hs.symbols.gmt and h.all.v2023.2.Hs.symbols.gmt, were sourced from the MSigDB database (|normalized enrichment score (NES)| > 1 and adj.*P <* 0.05). For examining pathway differences between groups with high and low biomarker expressions, Gene Set Variation Analysis (GSVA) was utilized (|t| > 2 and *P <* 0.05). Initially, differences between these groups were analyzed using the limma package. Subsequent GSVA were performed utilizing KEGG and Hallmark gene sets from the MSigDB database.

### Construction of regulatory networks for biomarkers

To further investigate the regulatory mechanism of disease biomarkers, the corresponding miRNAs of biomarkers and the lncRNAs corresponding to miRNAs were retrieved in the StarBasev2.0 database (http://starbase.sysu.edu.cn/). During the prediction of miRNAs, clipExpNum > 3 and pancancerNum > 0 were set, and for the prediction of lncRNAs, clipExpNum > 30 and pancancerNum > 5 were set. Finally, an lncRNA-miRNA-mRNA regulatory network was established, outlining the intricate regulatory pattern governing the expression of biomarkers. The transcription factors (TFs) of biomarkers were predicted in the ChEA3 database (https://amp.pharm.mssm.edu/ChEA3). The TFs with ChIP-seq data support in the ENCODE database (https://www.encodeproject.org/) were screened, and a network graph was plotted using the ggraph software.

### Drug prediction and molecular docking

To further predict potential targeted drugs for biomarkers, we utilized the DSigDB database (https://dsigdb.tanlab.org/) to identify target drugs associated with various biomarkers. Subsequently, we perform molecular docking on the highest scoring, non-toxic drugs from the DSigDB database results. Initially, we download the corresponding protein structures of the biomarkers from the PDB database and AlphaFold database, and acquire the 3D molecular structures of the corresponding compounds from the PubChem database. By using Autodock software, we perform molecular docking between biomarker protein structures and their respective compounds, and calculate the binding energy between the ligand and receptor. Generally, a more stable ligand-receptor conformation requires less binding energy. A binding energy of ≤ −5.0 kcal/mol suggests strong binding activity (*P* < 0.01)^24^.

### Human Corneal Lenticule Acquisition

This study was approved by the Ethics Committee of Hangzhou MSK Eye Hospital (Hangzhou, Zhejiang, China; ethics approval number: MSKLL250901) and conducted in accordance with the ethical principles of the Declaration of Helsinki. All corneal tissue donors underwent routine SMILE procedures. Written informed consent was obtained from each donor, or their legal guardians after a comprehensive explanation of the study’s nature and potential risks.

This study enrolled 20 patients (aged 18–24 years, only one eye per patient was included) scheduled for SMILE surgery, including 10 eyes with low myopia (≥ −3.00 D and < 0 D) and 10 eyes with high myopia (≤ −6.00 D). All patients underwent routine SMILE procedures with successful extraction of corneal stromal lenticules. From each group, six lenticules were allocated for mRNA analysis and immediately preserved in RNA stabilization solution, while the remaining four lenticules designated for protein quantification were directly snap-frozen in empty cryovials.

### Quantitative reverse transcription PCR (RT-qPCR)

Total RNA was isolated using the FastPure Complex Tissue/Cell Total RNA Isolation Kit (Vazyme, Nanjing), and its purity was assessed with a Nano-500 micro-spectrophotometer to ensure high-quality RNA for downstream applications. Complementary DNA (cDNA) synthesis was carried out using the ABScript III RT Master Mix with gDNA Remover (RK20429, ABclonal, Wuhan) following the manufacturer’s protocol. RT-qPCR was performed using the Genious 2X SYBR Green Fast RT-qPCR Mix (RK21205, ABclonal, Wuhan). Primer sequences used in the experiments are detailed in Supplementary Table 1. GAPDH served as the internal reference gene, and relative gene expression levels were determined using the ^2−ΔΔCt^ method.

### Western blot

Total protein extracts were prepared using RIPA buffer (Beyotime, Shanghai, China). Protein concentrations were measured using the BCA Protein Assay Kit (Beyotime, Shanghai, China) following the manufacturer’s instructions. The protein extracts were then mixed with 5× protein loading buffer (Servicebio, Beijing, China) in a 4:1 ratio, denatured at 95 ℃ for 10 min in a metal bath, and stored at either − 20 ℃ or −80 ℃ for subsequent analysis. For SDS-PAGE electrophoresis, separating and stacking gels were prepared based on the molecular weight of the target proteins. Stacking gels were run at 80 V for 30–40 min, followed by separating gels at 120 V until the pre-stained protein marker reached the bottom of the gel. Proteins were then transferred onto PVDF membranes in an ice bath under a constant current of 200 mA for 1 h. For immunoblotting, the membranes were rinsed with TBST buffer and blocked with 5% skimmed milk powder for 30 min to prevent nonspecific binding. Primary antibodies were diluted according to manufacturer recommendations and incubated with the membranes overnight at 4℃. Secondary antibodies were diluted at a ratio of 1:5000 and incubated at room temperature for 30 min. Finally, the membranes were treated with ECL substrate solution and visualized using a chemiluminescence imaging system.

### Statistical analysis

All analyses were executed in R software (v 4.2.2). Differences between groups were analyzed by Wilcoxon test. *P <* 0.05 was considered statistically significant.

## Results

### Identification and enrichment analysis of DEGs and candidate genes

**Fig. 1:**
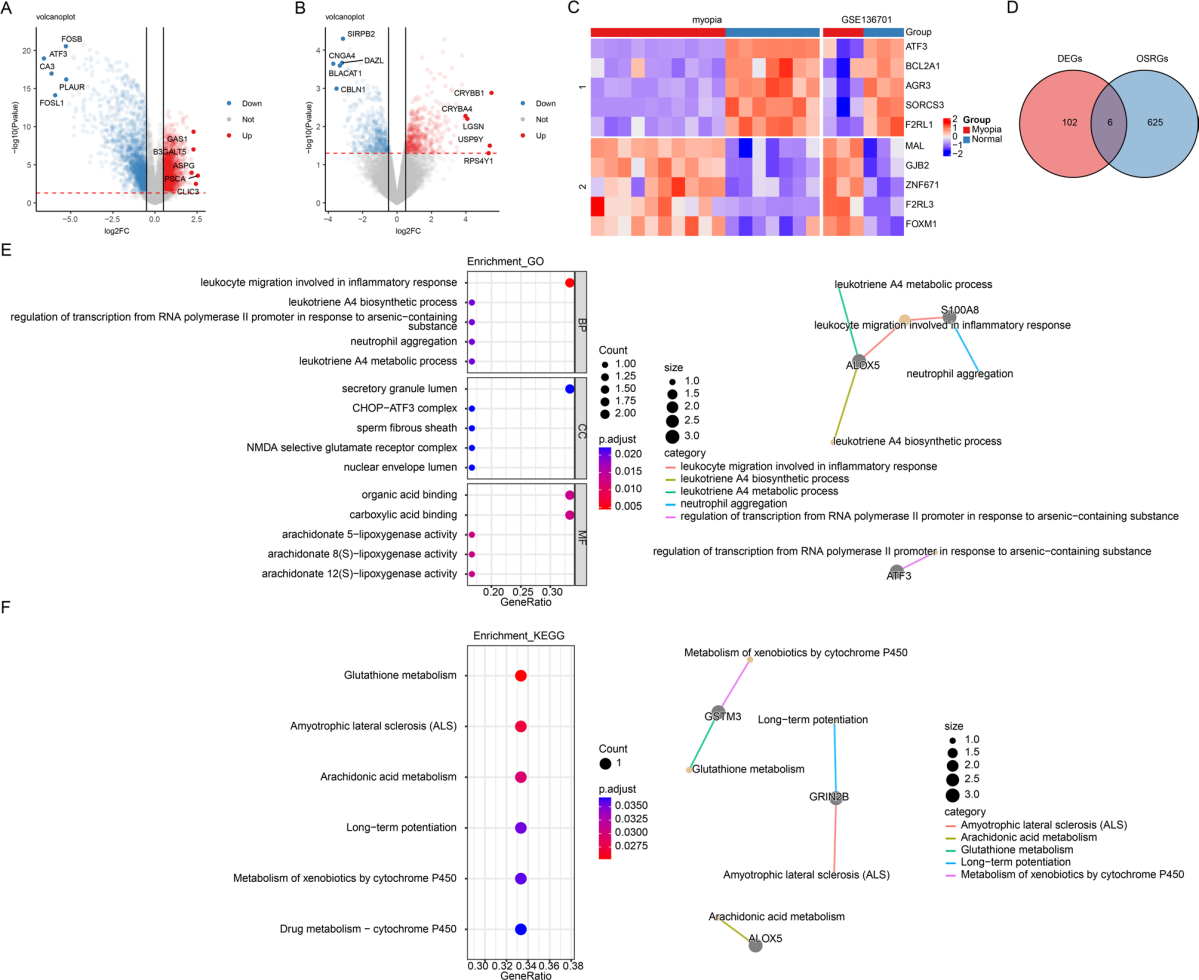
Identification and analysis of candidate genes. (A) Volcano plot of differentially expressed genes (DEGs) in the merger dataset. (B) Volcano plot of differentially expressed genes (DEGs) in the GSE136701. (C) Heatmap of Top5 differential gene expression. This map was generated using R version 4.2.2 (https://www.r-project.org/) with the ComplexHeatmap package version 2.14.0 (https://github.com/jokergoo/ComplexHeatmap). (D) Venn plot of DEGs and oxidative stress-related genes (OSRGs) to identify candidate genes. (E-F) Gene Ontology (GO) and Kyoto Encyclopedia of Genes and Genomes (KEGG) pathway analyses highlight the biological functions and signaling pathways enriched among candidate genes (Kanehisa Laboratories, KEGG Pathway Database; https://www.kegg.jp/kegg/pathway.html).

Through differential expression analysis, merge dataset revealed 3,924 DEGs between myopia and control samples, with 2,060 upregulated and 1,864 downregulated genes (Fig. 1A and C). In the GSE136701 dataset, 908 DEGs were identified, including 462 upregulated and 446 downregulated genes (Fig. 1B and C). By integrating the results from both analyses, a total of 108 DEGs were consistently identified, comprising 51 upregulated and 57 downregulated genes. The enrichment analysis revealed the potential biological functions and pathways of these DEGs. GO analysis identified a total of 636 terms, with significant enrichment in 470 terms related to BP, 47 terms related to CC, and 119 terms related to MF, such as regulation of peptidase activity, postsynaptic membrane, and peptidase regulator activity (Supplementary Fig. 1 A). Additionally, KEGG analysis highlighted four significantly enriched pathways, axon guidance, taurine and hypotaurine metabolism, glutathione metabolism, and biotin metabolism (Supplementary Fig. 1B). Next, a total of 6 candidate genes were obtained by overlapping OSRGs and 108 DEGs (Fig. 1D). The six candidate genes were enriched in a total of 159 GO terms, comprising 114 BP terms, 7 CC terms, and 38 MF terms. Specifically, these included leukocyte migration involved in inflammatory response, secretory granule lumen, and organic acid binding (Fig. 1E). In the KEGG analysis, six pathways were enriched, such as glutathione metabolism, and arachidonic acid metabolism (Fig. 1F).

### ATF3, GRIN2B, and GSTM3 identified as biomarkers

**Fig. 2:**
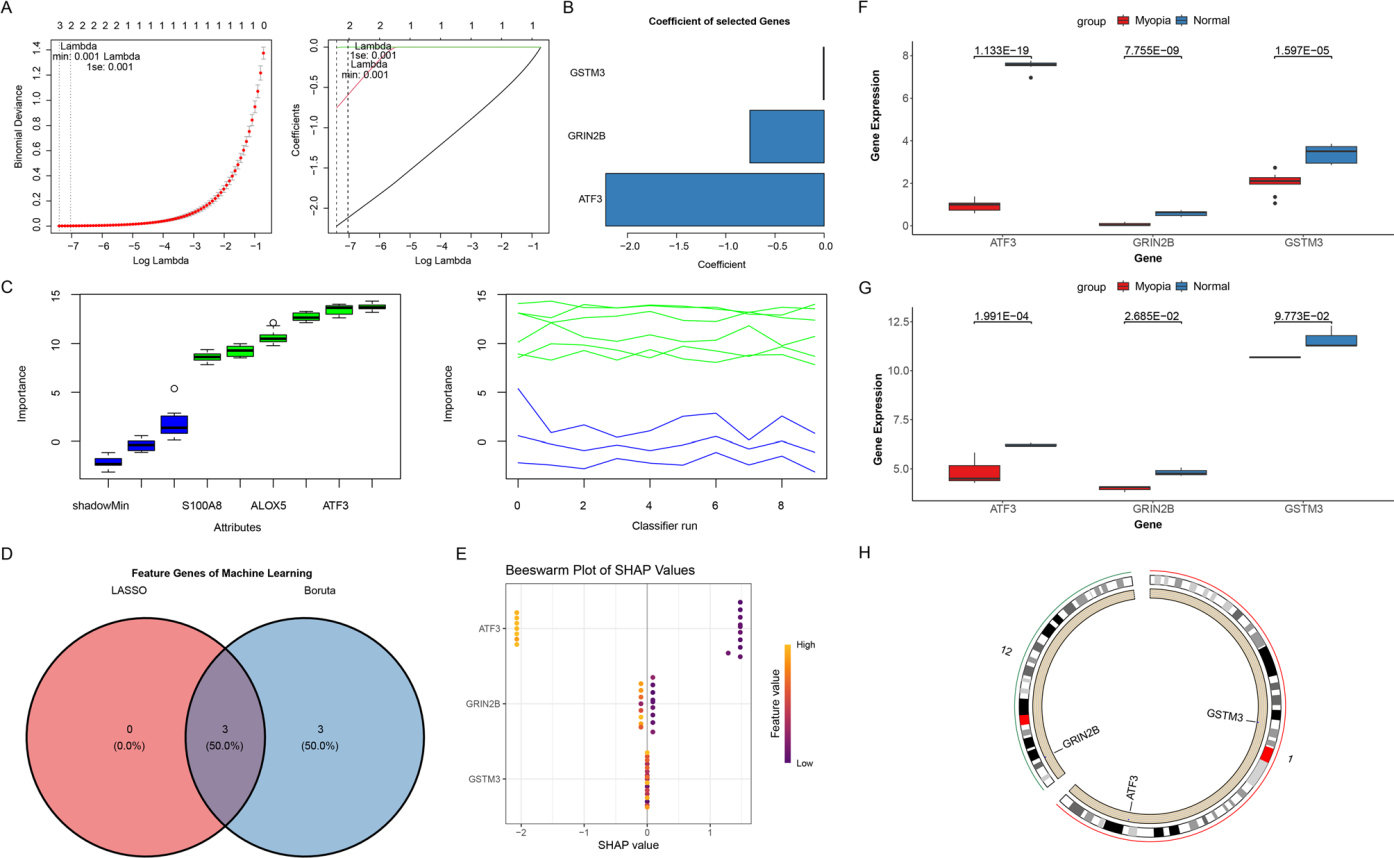
Identification and analysis of biomarkers. (A) Least Absolute Shrinkage and Selection Operator (LASSO) coefficient profiles. Genes from univariate Cox regression analysis were narrowed down by the lasso algorithm. (B) Characteristic gene coefficients. (C) Boruta screening for characterisation genes. (D) Venn diagrams for machine learning algorithms. (E) Beeswarm plot summarizing SHapley Additive exPlanations (SHAP) values for the top features in the prediction model. Each point represents an individual sample; the horizontal position indicates the SHAP value (contribution to the prediction outcome), with positive values denoting increased risk and negative values denoting decreased risk. Color intensity reflects the actual normalized expression level of the feature (light color: high expression; dark color: low expression). (F-G) Box plot of biomarker expression levels in merger dataset and GSE136701. (H) Chromosomal localisation of biomarkers.

The identification of diagnostic biomarkers for myopia was performed using a multi-step machine learning framework to enhance robustness and interpretability. First, LASSO regression selected three potential biomarkers with the following coefficients: ATF3 (−2.22), GRIN2B (−0.76), and GSTM3 (−0.01), where coefficient magnitude reflects relative feature importance (Fig. 2A, B). Second, Boruta algorithm confirmed all six candidate genes as relevant diagnostic features (Fig. 2C), with detailed importance metrics (mean, median, maximum, and minimum importance) provided in Supplementary Table 2. The intersection of LASSO and Boruta results consistently identified ATF3, GRIN2B, and GSTM3 as robust biomarkers (Fig. 2D). To address model interpretability and mitigate their black-box nature, we performed SHAP analysis. The results demonstrated that ATF3 was the predominant contributor to the prediction model (SHAP summary: Gain = 0.978, Cover = 0.954, Frequency = 0.929), while GRIN2B showed substantially lower but detectable importance (Gain = 0.022, Cover = 0.046, Frequency = 0.071), identifying ATF3 as the key driver in the prediction model (Fig. 2E). Differential analysis revealed that all three of these biomarkers exhibited low expression levels in myopia (*P <* 0.0001) (Fig. 2F, G). Given the consistent experimental validation and strong relevance identified in both LASSO and Boruta algorithms, GSTM3 was maintained in our narrative. Figure 2H presented their chromosomal localizations. However, MR analysis did not establish a causal relationship between the identified biomarkers and myopia risk (ATF3: *P =* 0.52; GSTM3: *P =* 0.29), and no genome-wide significant cis-eQTLs were available for GRIN2B, precluding its inclusion in the MR analysis. (Supplementary Fig. 1 C), suggesting that these biomarkers were not a direct contributor to the onset of myopia, but rather that it might be the onset of myopia that leads to altered gene expression.

### Establishment of a nomogram for myopia

**Fig. 3:**
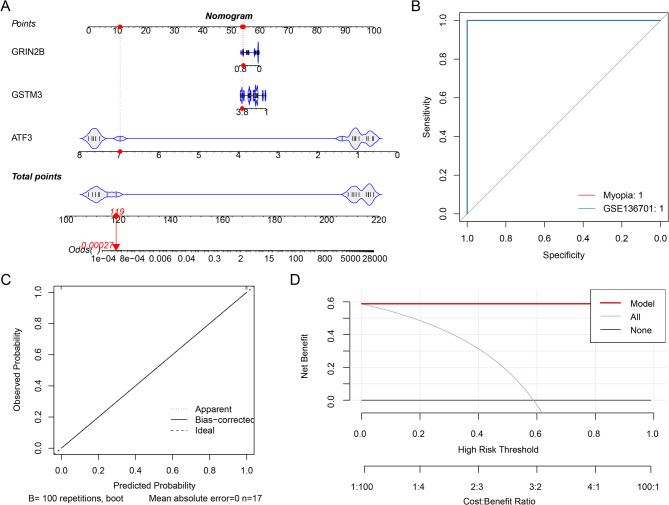
Nomogram based on biomarkers. (A) Nomogram. (B) Receiver Operating Characteristic (ROC) curve of the nomogram. (C) Calibration curve of the nomogram. (D) Decision curve analysis (DCA) of the nomogram.

Based on the obtained biomarkers, a nomogram for assessing myopia progression was established (Fig. 3A). Independent prognostic risk factors can be turned into a visual chart using the nomogram, which aids in individualized prognostic evaluation. The ROC curve indicated that the model’s area under the curve (AUC) value was 1 (Fig. 3B), indicating a highly effective diagnostic performance of the model. The calibration curve validated the efficacy of the nomogram model in predicting myopia progression (Fig. 3C). Additionally, a DCA was conducted to assess the clinical utility of the nomogram model. The DCA indicated that the nomogram provided a significant net benefit over a broad spectrum of threshold probabilities, demonstrating its strong predictive capability (Fig. 3D).

### Revealing the relationship between biomarkers and immune cells

**Fig. 4:**
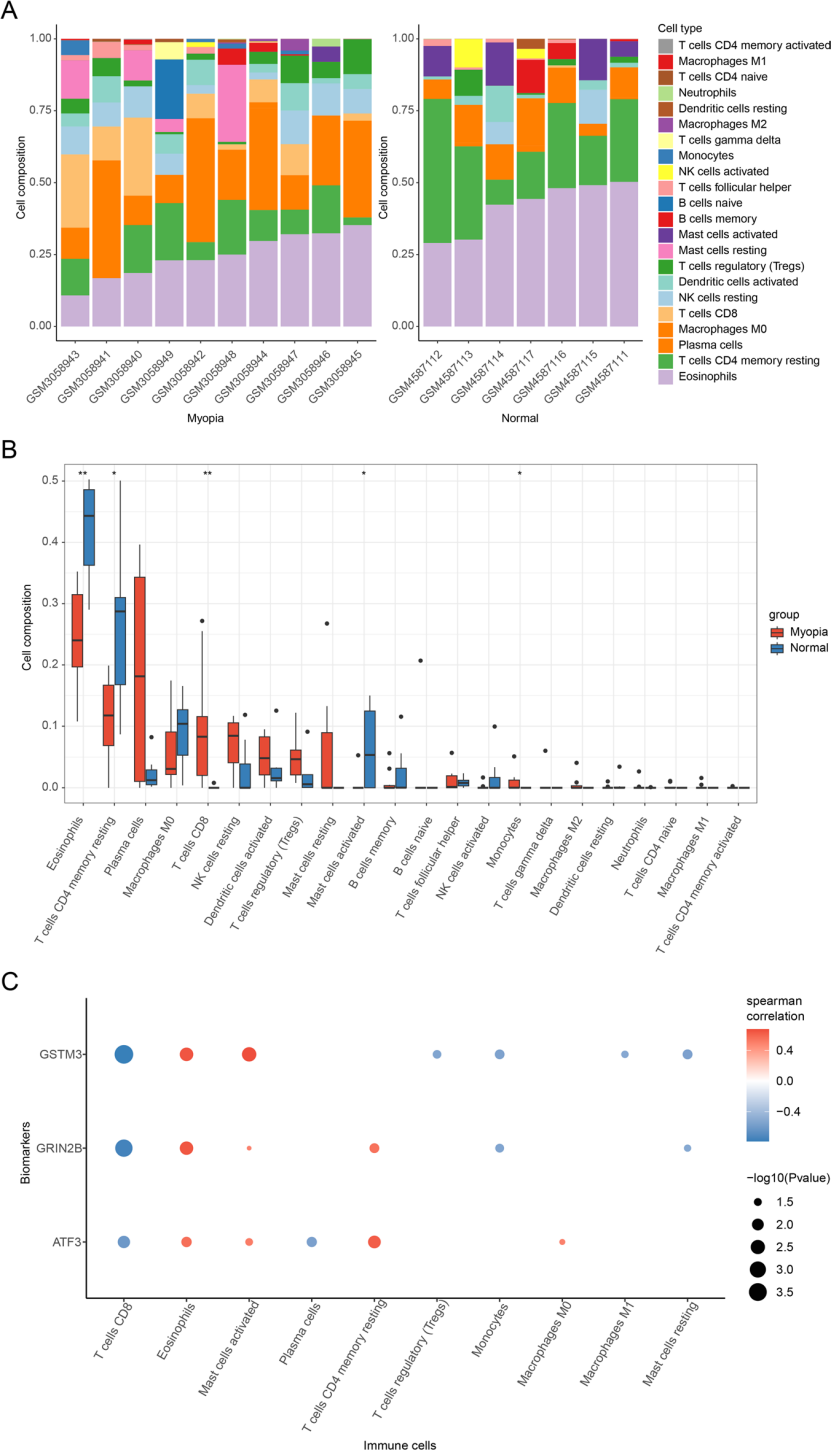
Immune cell infiltration analysis. (A) The relative abundances of 22 infiltrated immune cells between myopic and controls. (B) Boxplots showed the differences in immune infiltrating between myopic and controls. (C) Correlation analysis between biomarker expression levels and infiltrated immune cells.

The CIBERSORT algorithm was employed to investigate potential differences in the immune system between the myopia and control groups, providing a visual representation of the proportions of 22 infiltrating immune cell types (Fig. 4A). The results indicated significant differences in Eosinophils (*P* = 0.0046), memory resting CD4 + T cells (*P* = 0.025), CD8 + T cells (*P* = 0.0063), activated mast cells (*P* = 0.029), and monocytes (*P* = 0.040) among myopia patients (Fig. 4B). Correlation analysis between biomarkers and these immune cells revealed significant associations for 10 types of immune cells with at least one biomarker. Notably, CD8 + T cells showed strong negative correlations with all three biomarkers (ATF3: cor = −0.62, *P =* 0.008; GRIN2B: cor = −0.76, *P =* 0.00045; GSTM3: cor = −0.79, *P =* 0.00017), whereas eosinophils exhibited significant positive correlations (ATF3: cor = 0.57, *P =* 0.017; GRIN2B: cor = 0.65, *P =* 0.0048; GSTM3: cor = 0.65, *P =* 0.0046) (Fig. 4C). It is noticed that our correlation analysis does not establish cellular co-localization, but rather reflects coordinated changes in biomarker expression and immune cell infiltration within the corneal microenvironment. These findings suggested that OSRGs might regulate the infiltration of these immune cells, thereby influencing the progression of myopia.

### GSEA and GSVA of biomarkers

**Fig. 5:**
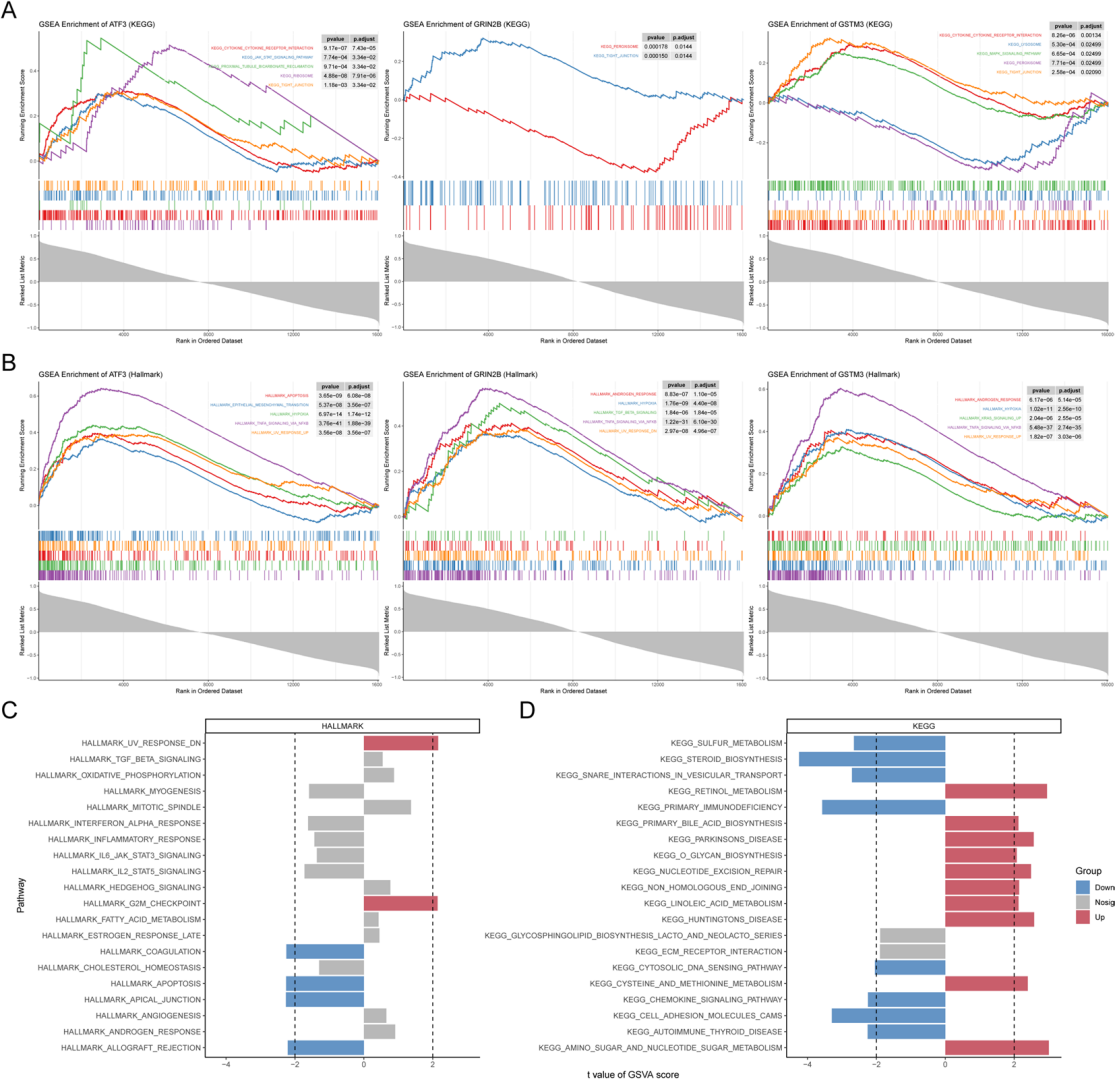
GSEA and GSVA enrichment analysis. (**A**) Gene Set Enrichment Analysis (GSEA) of biomarkers based on the KEGG pathway database. (**B**) GSEA analysis of biomarkers based on the Hallmark gene sets. (**C**) Gene Set Variation Analysis (GSVA) of biomarkers on the Hallmark gene sets. (**D**) GSVA analysis of biomarkers on KEGG.

To deepen our understanding of the relationship between biomarkers and their associated pathways, revealing potential mechanistic insights, enrichment analysis was conducted. The GSEA analysis revealed that ATF3, GRIN2B, and GSTM3 were associated with the tight junction pathway in KEGG and were commonly linked to UV response, TNFA signaling via NFKB, and hypoxia pathways in Hallmark (Fig. 5A, B). The GSVA results indicated significant differences in pathways such as steroid biosynthesis, primary immunodeficiency, and adhesion molecules cams within KEGG (Fig. 5C). In the Hallmark database, notable differences were observed in the apical junction, apoptosis, and coagulation pathways (Fig. 5D). These findings suggested that ATF3, GRIN2B, and GSTM3 may play crucial roles in cellular processes related to tight junctions and responses to environmental stressors such as UV exposure and hypoxia. The differential pathways identified by GSVA further imply potential mechanisms involving steroid biosynthesis and immune function that could be pivotal in understanding disease pathogenesis or therapeutic targets.

### Prediction of potential regulatory mechanisms

**Fig. 6:**
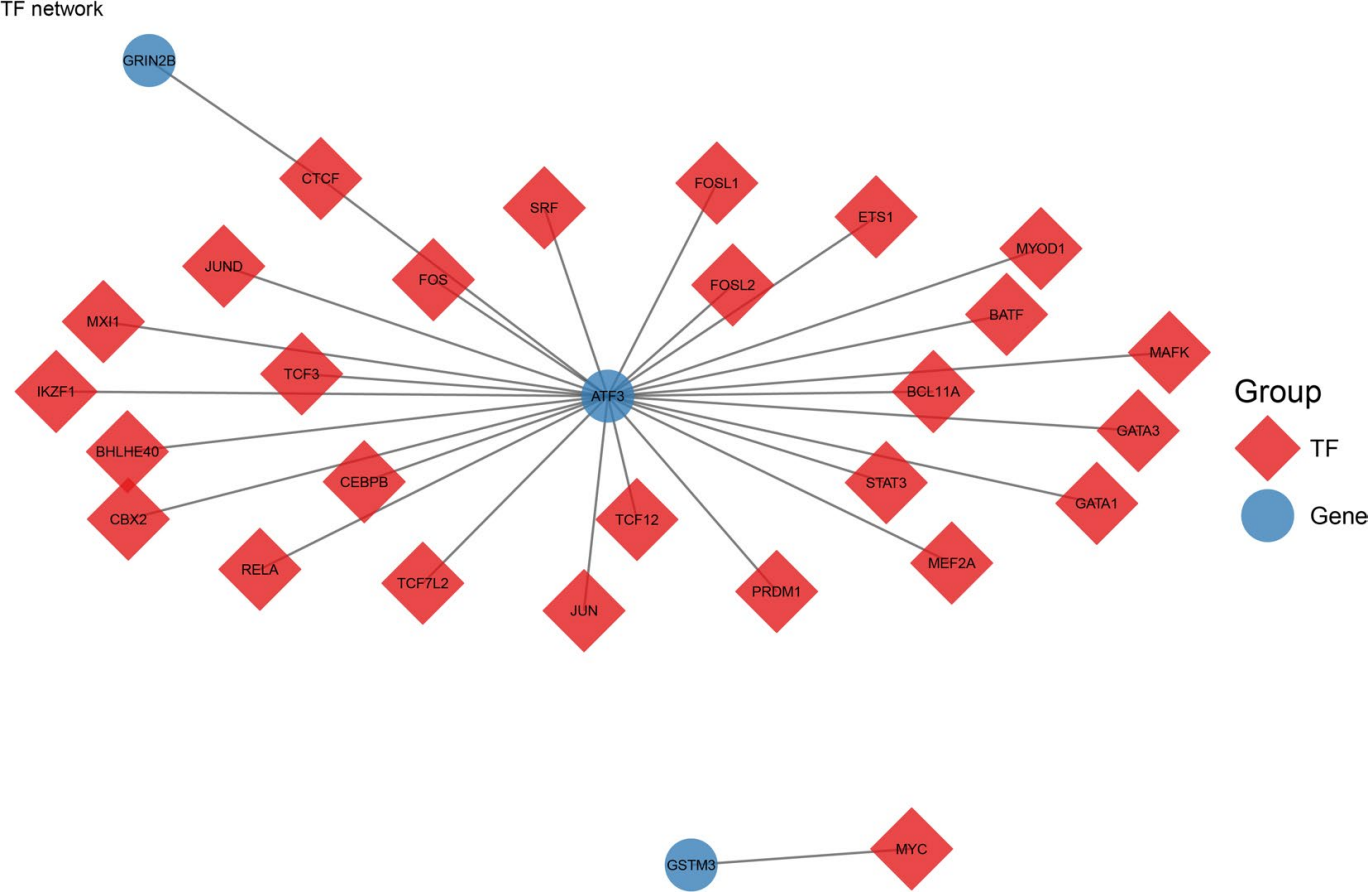
The predicted transcription factors (TFs) network of biomarkers. Red diamonds represent TFs and blue circles represent genes.

A total of 27 potential TFs were predicted, and an mRNA-TF interaction network was constructed. This network indicated that ATF3 was regulated by TCF12, and GSTM3 was regulated by MYC (Fig. 6). Additionally, a competing endogenous RNA (ceRNA) network integrating 45 miRNAs and 31 lncRNAs was established (79 nodes and 97 edges in total), highlighting key interactions including hsa-let-7a-5p and AC074117.1 for ATF3, hsa-miR-330-3p and NEAT1 for GRIN2B, as well as hsa-miR-26b-5p and NORAD for GSTM3 (the complete network data are provided in Supplementary Table 3).

### Prediction of targeted drugs for biomarkers

**Fig. 7:**
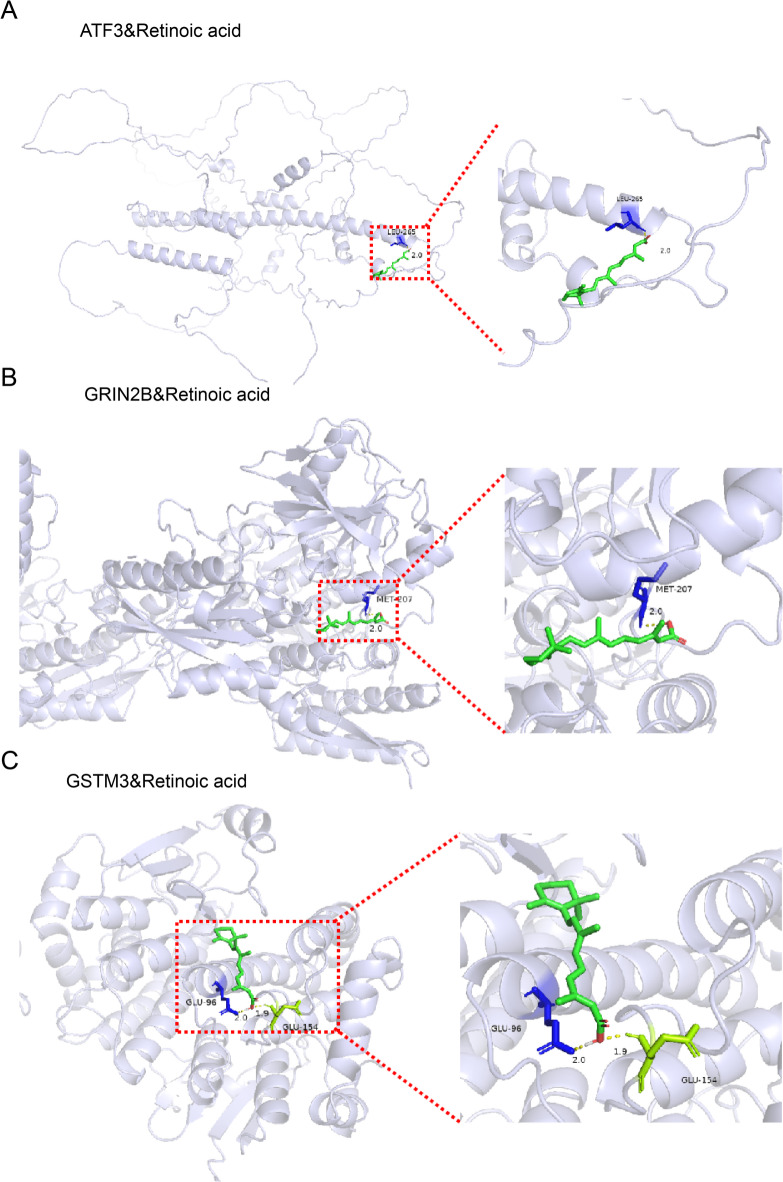
Molecular docking site map for Retinoic acted on the biomarkers. (**A**) ATF3. (**B**) GRIN2B. (**C**) GSTM3.

By employing the DSigDB database, we anticipated drugs for the biomarkers, gathered drug-marker interactions, and created a network that involved 3 biomarkers and 123 drugs. The top 10 candidate drugs ranked by combined score are listed in Table 1 (the full drug-biomarker interaction list is available in Supplementary Table 4), where Retinoic acid (RA) was identified as a common regulator for all three biomarkers. Docking was carried out between the drugs and the molecular targets that were predicted. (Fig. 7A-C; Table 2). It is noted that the results regarding the retinoic acid-biomarker docking studies in this research are solely based on computational simulations and predictions. Further investigation is warranted to substantiate these findings.

**Table 1:** Top 10 candidate drugs targeting the biomarkers.

Term	*P*.value	Adjusted.*P*.value	Combined.Score	Genes
Retinoic acid CTD 00006918	0.00964454236943559	0.0286086567440885	219193.012872312	GSTM3;GRIN2B; ATF3
GLYOXAL CTD 00006046	0.00164914273085112	0.0286086567440885	6403.33480835207	ATF3
dnqx TTD 00007689	0.00164914273085112	0.0286086567440885	6403.33480835207	GRIN2B
AR-A014418 CTD 00004251	0.00179897576875959	0.0286086567440885	5741.92128812022	ATF3
DL-Glutamic acid TTD 00007645	0.00194879387700138	0.0286086567440885	5196.5535115997	GRIN2B
proadifen CTD 00006614	0.00254791692627063	0.0286086567440885	3729.25330632509	ATF3
trimipramine MCF7 UP	0.00299710227697048	0.0286086567440885	3054.58854972563	ATF3
cloperastine MCF7 UP	0.00314680082428191	0.0286086567440885	2877.37168963486	ATF3
clotrimazole MCF7 UP	0.00314680082428191	0.0286086567440885	2877.37168963486	ATF3
salubrinal CTD 00004410	0.00314680082428191	0.0286086567440885	2877.37168963486	ATF3

**Table 2:** Docking binding energies of core target molecules.

Symbol	ID	Molecule Name	Affinity(kcal/mol)	Hydrogen bonds
ATF3	AF-Q9XZS8-F1-v4	Retinoic acid	−4.8	1
ATF3	AF-Q9XZS8-F1-v4	Proadifen	−3.8	1
GRIN2B	5EWJ	Retinoic acid	−7.7	1
GRIN2B	5EWJ	DL-Glutamic acid	−5.3	5
GSTM3	3GTU	Retinoic acid	−7.3	2
GSTM3	3GTU	L-methionine	−4.4	4

3.8 Expression of ATF3, GRIN2B, and GSTM3 in corneal stromal tissue from patients with high and low myopia.

**Fig. 8:**
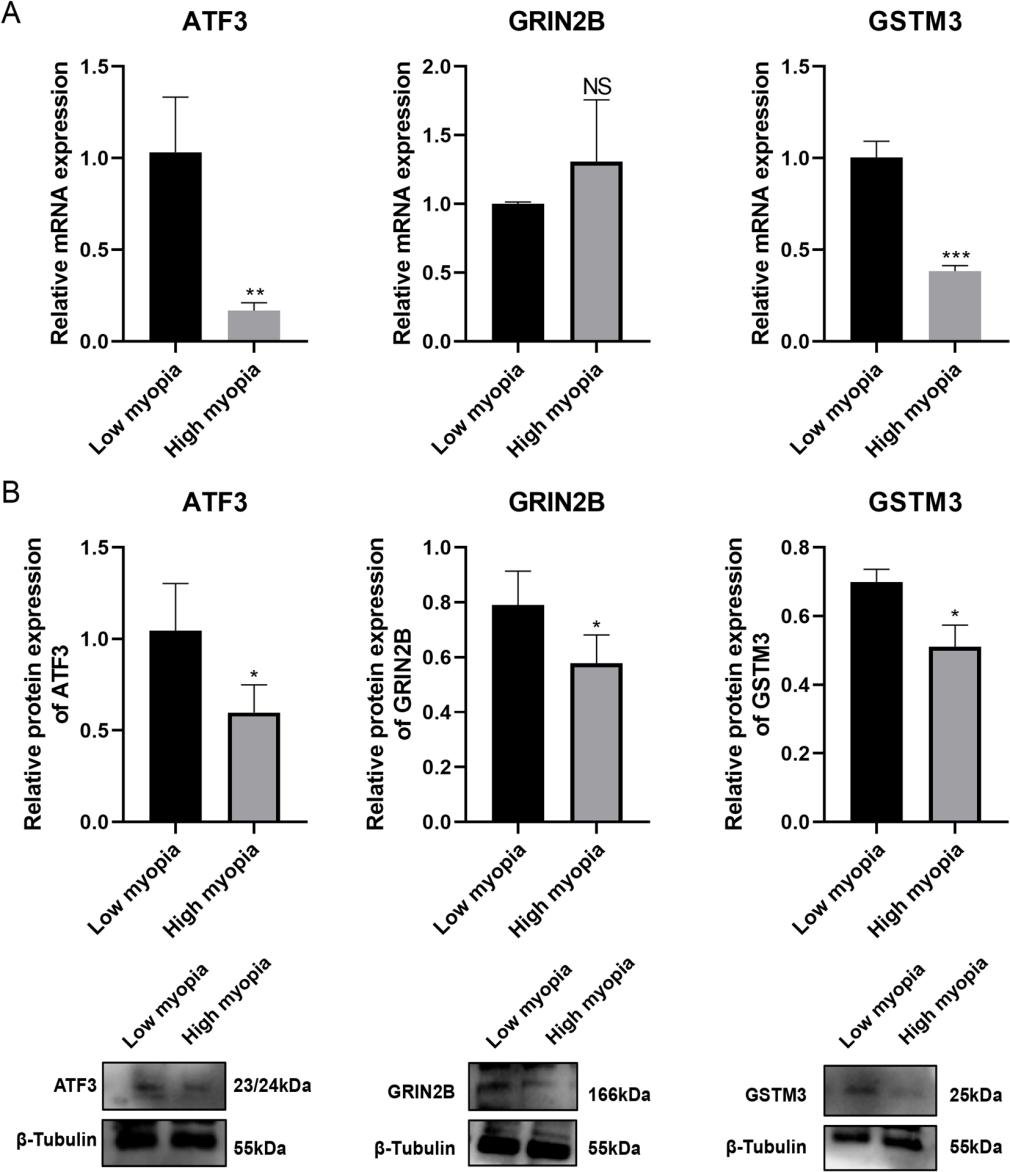
Expression levels of these biomarkers were examined in corneal stromal tissues obtained from patients undergoing SMILE surgery. (A) Quantitative reverse transcription PCR (RT-qPCR) (*n* = 6 per group) for mRNA expression levels of three biomarkers. (B) Western blot (*n* = 4 per group) for protein expression levels of three biomarkers. NS, not significant. *P < 0.05, **P < 0.01, ***P < 0.001. Low myopia: ≥ −3.00 D and < 0 D); high myopia: ≤ −6.00 D).

High myopia group and low myopia group comprised 10 and 10 eyes, respectively. Preoperative characteristics by group are listed in Table 3. There were no statistically significant differences between the two groups as regards the patient’s sex, age, and mean corneal power, allowing for the detection of the three biomarkers in the corneal tissue free from interference (*P >* 0.05). Analysis of mRNA levels in the extracted lenticules revealed significant downregulation of ATF3 (*P =* 0.0079) and GSTM3 (*P =* 0.0003) in the high myopia group compared to the low myopia group (*P =* 0.30) (Fig. 8A). However, the GRIN2B expression was too low for reliable quantitative analysis. At the protein level, all three biomarkers showed significant reduction in the high myopia group (ATF3: *P =* 0.030, GRIN2B: *P =* 0.042, and GSTM3: *P =* 0.011) (Fig. 8B).

**Table 3:** Preoperative characteristics of both groups.

Characteristic	Low Myopia (*n* = 10)	High Myopia (*n* = 10)	t	*P*-value
Sex (M/F)	4/6	5/5	-	-
Age (years)	19.7 ± 1.64	20.2 ± 2.10	−0.59	0.56
Sphere (D)	−2.50 ± 0.53	−7.10 ± 0.73	16.18	< 0.001
Cylinder (D)	−0.43 ± 0.35	−0.63 ± 0.29	1.37	0.19
Axial Length (mm)	25.111 ± 0.53	27.146 ± 0.66	−7.63	< 0.001
Mean Corneal Power (D)	43.569 ± 0.75	43.893 ± 0.58	−1.09	0.29
Operation	SMILE	SMILE	-	-

## Discussion

The prevalence of myopia remains high worldwide, yet its pathogenesis is still not fully understood. Existing research has indicated a link between oxidative stress and the progression of myopia, with studies already conducted on the aqueous humor, retina, and lens^25^. Moreover, minor changes in the cornea can potentially have significant impacts on the refractive power of the eye, possibly contributing to the development of myopia^26^. However, the role of the cornea in oxidative stress-involved myopia formation has not been explored. This study employs machine learning to preliminarily investigate the involvement of the cornea in the association between oxidative stress and myopia formation.

Using the machine learning algorithm, we identified ATF3, GRIN2B, and GSTM3 as potential corneal biomarkers associated with myopia, likely expressed in corneal epithelial cells, stromal keratocytes, and endothelial cells^27–29^. And meanwhile, SHAP visualizations revealed that ATF3 and GRIN2B consistently exhibited the highest SHAP values, identifying them as key drivers in the prediction model, suggesting that stress response in epithelial and stromal cells (ATF3) and glutamate signaling in epithelial and endothelial cells (GRIN2B) may play pivotal roles in corneal involvement in myopia. ATF3(Activating transcription factor 3) is a member of the ATF/CREB (cAMP-responsive element-binding protein) transcription factor family and plays a pivotal role in cellular stress responses. Its expression is significantly upregulated in response to harmful stimuli such as oxidative stress and endoplasmic reticulum stress. Previous studies have demonstrated that ATF3 expression is significantly upregulated during oxidative stress in cardiovascular diseases and vascular dementia, initiating inflammatory cascades that result in vascular endothelial cell damage^30^. Additionally, ATF3 has been shown to exhibit a negative correlation with activity of sodium-potassium ATPase^31,32^. GRIN2B encodes a subunit of the N-methyl-D-aspartate (NMDA) receptor, an ionotropic glutamate receptor essential for synaptic transmission and plasticity in the central nervous system. Although traditionally studied in neurons—where its upregulation is linked to oxidative stress in Alzheimer’s-like models^33^—emerging evidence supports its legitimate expression in ocular tissues. For instance, NMDA receptors containing GRIN2B have been identified in corneal endothelial cells^34^, and the corneal epithelium in human and rat expresses key components of glutamate signaling, including transporters (EAATs), the cystine/glutamate antiporter xCT, and glutathione synthetase^35^. The presence of xCT indirectly suggests that corneal cells may establish a localized glutamate-rich microenvironment capable of activating NMDA receptors. Thus, GRIN2B expression in the cornea is physiologically relevant and may participate in cellular stress response or tissue homeostasis. GSTM3 (Glutathione S-transferase mu 3) belongs to GSTs superfamily, which mainly catalyze the connection of glutathione to a variety of electrophilic chemical compounds, involved in the metabolism of various bioactive compounds^36^. GSTM3 exhibits strong antioxidative properties. Oxidative stress in the lens plays a significant role in the development of age-related cataracts (ARC). Studies have revealed that the expression of GSTM3 is downregulated during the progression of ARC^37^. While direct comparative studies on ocular GSTM3 expression are limited, existing literature reports substantial variation in GSTM3 tissue distribution and expression levels across mammalian species^38^. Our study provides preliminary evidence linking the expression of three biomarkers in human corneal tissues to myopia progression, with the cellular context (epithelial, stromal, and endothelial cells) informing the functional interpretation of oxidative stress and signaling pathways in corneal involvement. A counterintuitive finding was the reduced ATF3 expression in high myopic corneas, contrary to its established role in oxidative stress, which may reflect stress response or cell loss in epithelial and stromal keratocytes. We propose several non-exclusive explanations: (1) prolonged antioxidant depletion in a chronic stress milieu; (2) attrition of responsive cells through apoptosis; or (3) inhibitory crosstalk from other signaling pathways. Separately, the detection of GRIN2B in the human corneal stroma reveals previously unrecognized signaling complexity within this tissue, potentially associated with myopia development. The decreased expression of GSTM3 in our highly myopic corneal tissues suggests a compromised antioxidant defense capacity. Further experimental investigations, such as immunohistochemistry or in situ hybridization, are required to confirm the cell-specific expression and functional roles of these biomarkers in the cornea.

The results of the enrichment analysis suggest that the pathways associated with the three biomarkers, ATF3, GRIN2B, and GSTM3, are involved in processes related to tight junctions, hypoxia, and ultraviolet (UV) exposure. Previous studies have demonstrated that hypoxia occurs in scleral tissue during the development and progression of myopia, leading to macroscopic structural changes in the sclera and ultimately contributing to the formation of myopia^39^. The exact cause of hypoxia in the sclera remains unclear, however, both the sclera and cornea are supplied by the ciliary artery system^40^. The tight junctions formed between adjacent corneal endothelial cells constitute the corneal-aqueous barrier, which effectively prevents the penetration of aqueous humor into the cornea. Studies have found that when the cornea experiences hypoxia, the expression of the tight junction protein ZO-1 in corneal endothelial cells is significantly reduced, leading to the disruption of the corneal-aqueous barrier^41^. Furthermore, studies have demonstrated that ultraviolet (UV) irradiation can induce alterations in corneal tissue at the genetic, molecular, and cellular levels^42^. Consequently, when the corneal microenvironment undergoes changes, the corresponding responses to UV-induced damage may also vary significantly.

The involvement of immune mechanisms in myopia pathogenesis is well recognized, with both innate and adaptive immunity contributing to disease progression^43,44^. Innate immune cells, such as neutrophils, macrophages, and eosinophils, play a role in tissue remodeling, debris clearance, and the creation of extracellular traps. Adaptive immunity, mediated by T cells and B cells, regulates inflammatory processes through cytokine and chemokine secretion, cell differentiation, and maintenance of immune tolerance. These coordinated immune activities ultimately drive key pathological features of myopia through a cascade of tissue-level changes, including extracellular matrix (ECM) remodeling, scleral elongation, and choroidal thinning^44^. Our immune infiltration analysis provided direct cellular evidence for this notion, revealing a significant dysregulation of the immune landscape in myopia patients. Specifically, we observed an increase in CD8 + T cells and monocytes, accompanied by a decrease in eosinophils and resting memory CD4 + T cells. This cellular profile aligns with and extends previous findings of upregulated immune-related pathways in myopia models^43^, further reinforces the central role of the immune system in myopia pathogenesis. The observed decrease in eosinophils suggests a potential immunological shift characterized by enhanced Th1-type responses alongside suppressed Th2-type immunity. This polarization is further reinforced by the elevated levels of CD8 + T cells and monocytes, both known for their pro-inflammatory roles in immune activation and tissue remodeling^45,46^. Further detection and analysis of the characteristic Th1/Th2 cytokine ratio (IFN-γ/IL-4) are required.

For the analytical results between the three downregulated biomarkers and key immune cell populations: all three genes showed negative correlations with CD8 **+** T cells and positive correlations with eosinophils. This consistent pattern suggests a potential coordinated relationship between the expression of these biomarkers and the altered immune landscape in myopia. To date, no studies have directly documented the expression of ATF3, GRIN2B, or GSTM3 in CD8 + T cells, CD4 + T cells, or eosinophils within corneal tissue. However, evidence from other physiological contexts supports their functional relevance in immune cells. For instance, in tumor microenvironments, exhausted CD8 + T cells display an increased binding motif for the ATF3 transcription factor following treatment with a combination of decitabine and PD-1 inhibitors^14^. Additionally, elevated ATF3 expression in CD4 + helper T cells has been shown to participate in the differentiation of follicular helper T cells (Tfh)^47^. These findings collectively suggest that these biomarkers are not only expressible in immune cells but may also play functional roles in immune regulation. Subsequent studies should use immunohistochemistry to quantify CD8 + T cells and eosinophils and assess their co-expression of the three biomarkers in myopic corneas.

As the biologically active metabolite of vitamin A (retinol), RA has been shown to play a critical role in maintaining adult corneal homeostasis and regeneration^48,49^. Deficiency in RA synthesis has been associated with corneal thinning, characterized by reduced stromal thickness, impaired corneal epithelial cell proliferation, and increased apoptosis^50^. Our study demonstrates that all three identified biomarkers exhibit molecular docking capability with RA, but the interventional role of retinoic acid in myopia progression hinges on elucidating the functions of these three biomarkers, mandating additional experimental studies.

This study has several limitations that should be considered when interpreting the results. First, the scarcity of large-scale myopia-specific corneal transcriptomic datasets necessitated the integration of corneal tissue-derived data for the discovery set and lens-derived data for validation, potentially masking some cornea-specific expression changes and introducing confounding factors. We fully acknowledge this as a recognized limitation in the field of bioinformatics studies on myopia. This limitation is further reflected in the absence of Mendelian randomization evidence for GRIN2B, which primarily stems from data availability constraints in current genomic resources rather than biological irrelevance. Second, the perfect discriminative performance (AUC = 1.000) of our nomogram likely indicates overfitting due to the limited sample size (*n* = 17), requiring validation in larger independent cohorts. In addition, key clinical and demographic confounders, including contact lens use, corneal thickness or biomechanics, age, ethnicity, and refractive history, were not accounted for due to incomplete metadata in public datasets and the limited sample size. These unmeasured variables may influence corneal gene expression and immune microenvironment features, thereby potentially limiting the generalizability of our findings and underscoring the need for future validation in larger and more diverse populations. Third, while we employed multiple interpretability methods to address the black box nature of machine learning, the biological mechanisms underlying the selected biomarkers and their observed correlations with immune cells (e.g., CD8 + T cells and eosinophils) remain computationally derived and require experimental confirmation. Fourth, biomarker expression was evaluated at a single time point in human corneal tissues, thus lacking data on temporal dynamics. Fifth, our focus on the human corneal stroma leaves expression patterns in other corneal layers unexplored. Finally, the therapeutic potential of the predicted drug-biomarker interactions, particularly involving retinoic acid, remains theoretical without experimental validation through binding assays or animal models.

To address these limitations, we have formulated a comprehensive research plan. First, we will validate the nomogram in larger, independent cohorts and conduct immunofluorescence co-staining experiments in animal models to examine the spatial relationships between biomarkers (ATF3, GRIN2B, GSTM3) and immune cells. Second, we will employ genetic approaches including knockout and overexpression models to functionally characterize these biomarkers and assess their impact on immune cell infiltration and cytokine profiles. Third, we plan to establish a multi-center “Myopic Corneal Biobank” to enable large-scale biomarker validation and develop point-of-care detection kits for clinical translation. These initiatives will help overcome the current data scarcity issues and provide more reliable, tissue-specific conclusions in future research. Fourth, temporal dynamics of biomarker expression will be investigated using lens-induced myopia models with varying induction durations, combined with detailed microscopic evaluation of corneal endothelial barrier structure and function to test our oxidative stress hypothesis. Changes in corneal refractive power will be assessed by analyzing correlations between corneal curvature, axial length, and total ocular refractive power. Finally, the predicted drug-target interactions will be systematically validated through in vitro binding assays and in vivo efficacy studies.

These coordinated efforts will help bridge the gap between computational predictions and biological validation, ultimately enhancing our understanding of myopia pathogenesis and facilitating the development of targeted therapeutic strategies.

## Conclusion

In summary, this study systematically integrated multiple bioinformatics approaches—including differential expression analysis, enrichment analysis, immune infiltration profiling, drug sensitivity prediction, GSEA/GSVA analyses, transcription factor prediction, mRNA-miRNA-lncRNA network construction, and Mendelian randomization—to systematically identify the potential roles and mechanistic associations of OSRGs (ATF3, GRIN2B, and GSTM3) in myopia pathogenesis. The findings provide supportive evidence linking corneal oxidative stress–related gene alterations to myopia development, offering a foundation for further research into diagnostic, preventive, and therapeutic strategies.

## Supplementary Information

Below is the link to the electronic supplementary material.

Supplementary Material 1

Supplementary Material 2

Supplementary Material 3

Supplementary Material 4

Supplementary Material 5

Supplementary Material 6

Supplementary Material 7

Supplementary Material 8

## Data Availability

The myopic datasets GSE112155, GSE151631, and GSE136701 analyzed in this study were collected from the GEO database (https://www.ncbi.nlm.nih.gov/geo/).
