# Correction: Gene expression and machine learning techniques uncover corneal biomarkers associated with oxidative stress in the myopia progression

**DOI:** 10.1038/s41598-026-50292-w

**Published:** 2026-05-08

**Authors:** Qingyi Zhou, Mingxia Ye, Zhiyong Zhang, Hongbo Luo, Yonggang Zhang, Hailan Zhao

**Affiliations:** 1 Department of Ophthalmology, Zhejiang Provincial People’s Hospital (Affiliated People’s Hospital), Hangzhou Medical College, Hangzhou, Zhejiang China; 2 Hangzhou MSK Eye Hospital, Hangzhou, Zhejiang China

Correction to: *Scientific Reports* 10.1038/s41598-026-46896-x, published online 30 March 2026

The original version of this Article contained an error in the order of the figures, where Figures 5, 6, 7 and 8 were published as Figures 7, 5, 8, and 6, respectively. As a result, the figure legends accompanied the incorrect figures.

The original Figures 5, 6, 7 and 8 and their accompanying legends appear below.

**Fig. 5:**
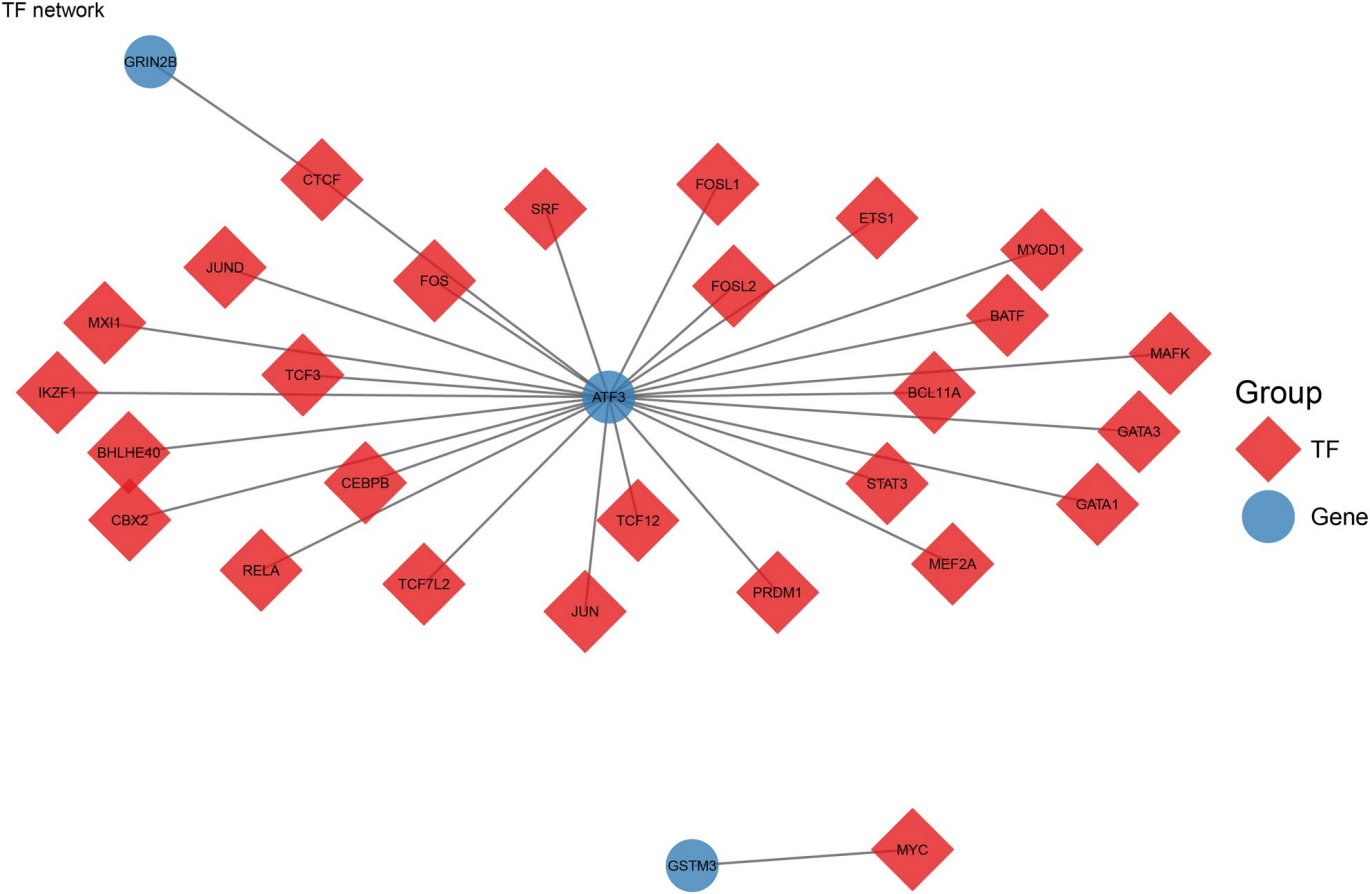
GSEA and GSVA enrichment analysis. (**A**) Gene Set Enrichment Analysis (GSEA) of biomarkers based on the KEGG pathway database. (**B**) GSEA analysis of biomarkers based on the Hallmark gene sets. (**C**) Gene Set Variation Analysis (GSVA) of biomarkers on the Hallmark gene sets. (**D**) GSVA analysis of biomarkers on KEGG.

**Fig. 6:**
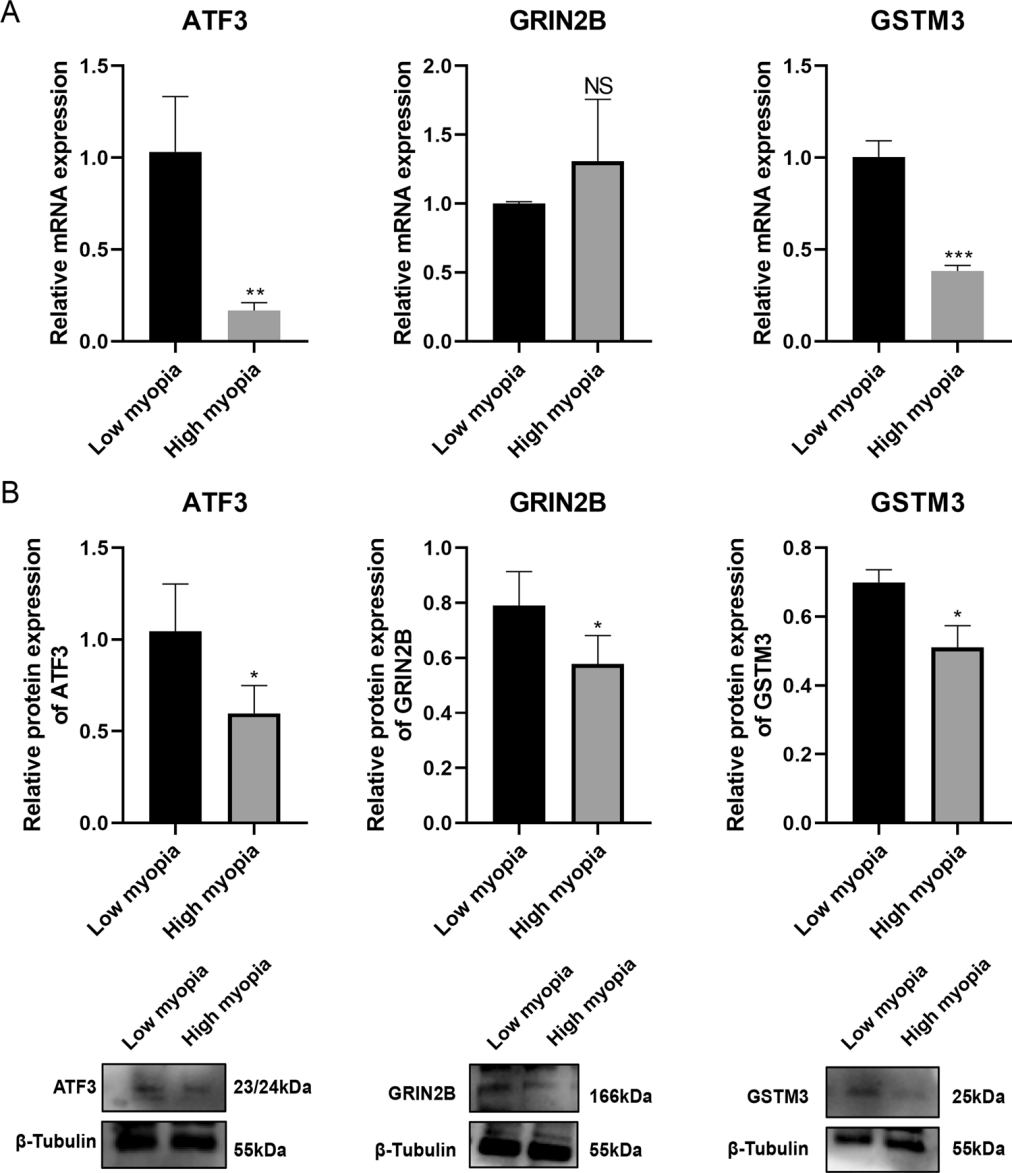
The predicted transcription factors (TFs) network of biomarkers. Red diamonds represent TFs and blue circles represent genes.

**Fig. 7:**
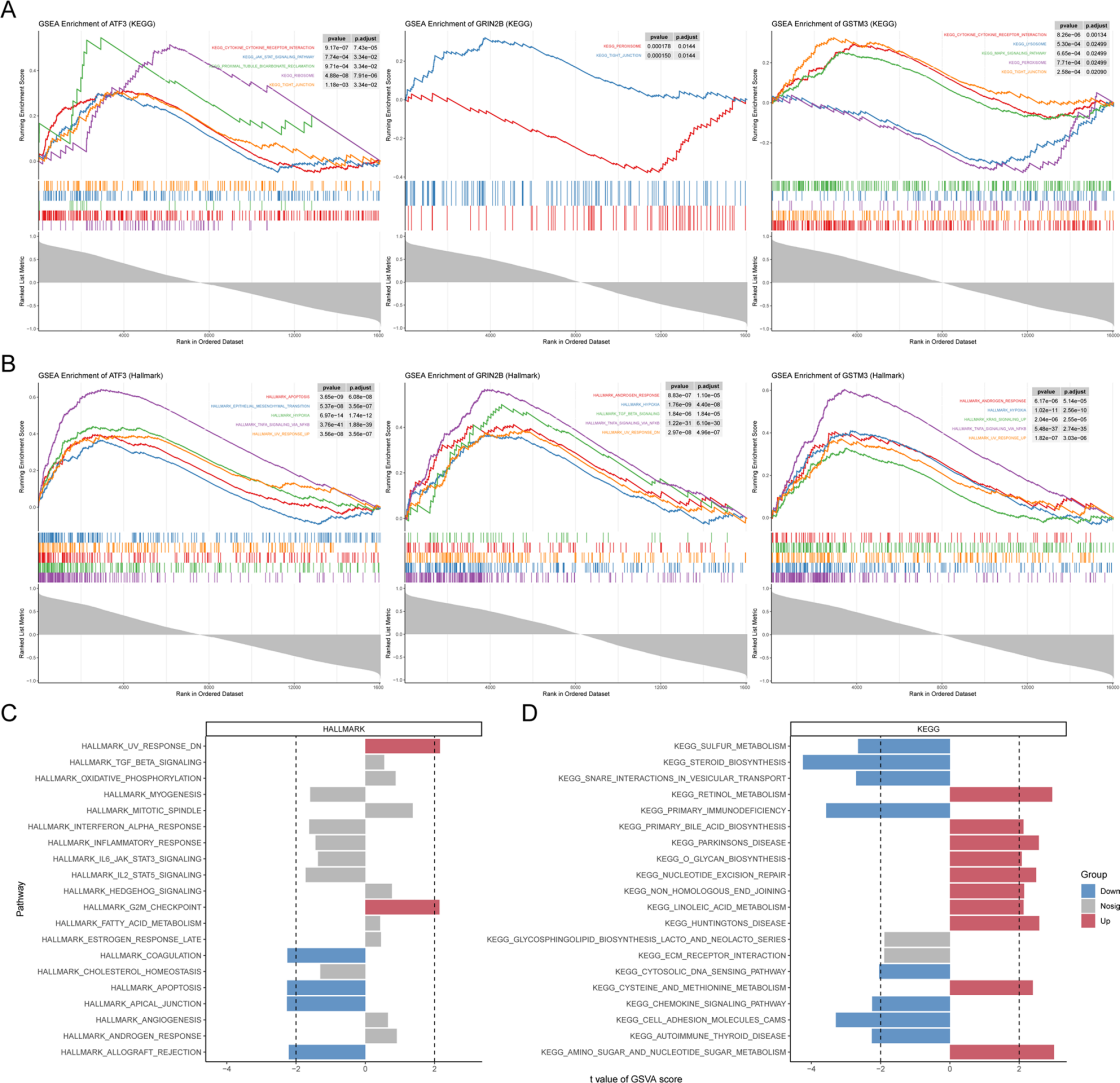
Molecular docking site map for Retinoic acted on the biomarkers. (**A**) ATF3. (**B**) GRIN2B. (**C**) GSTM3.

**Fig. 8:**
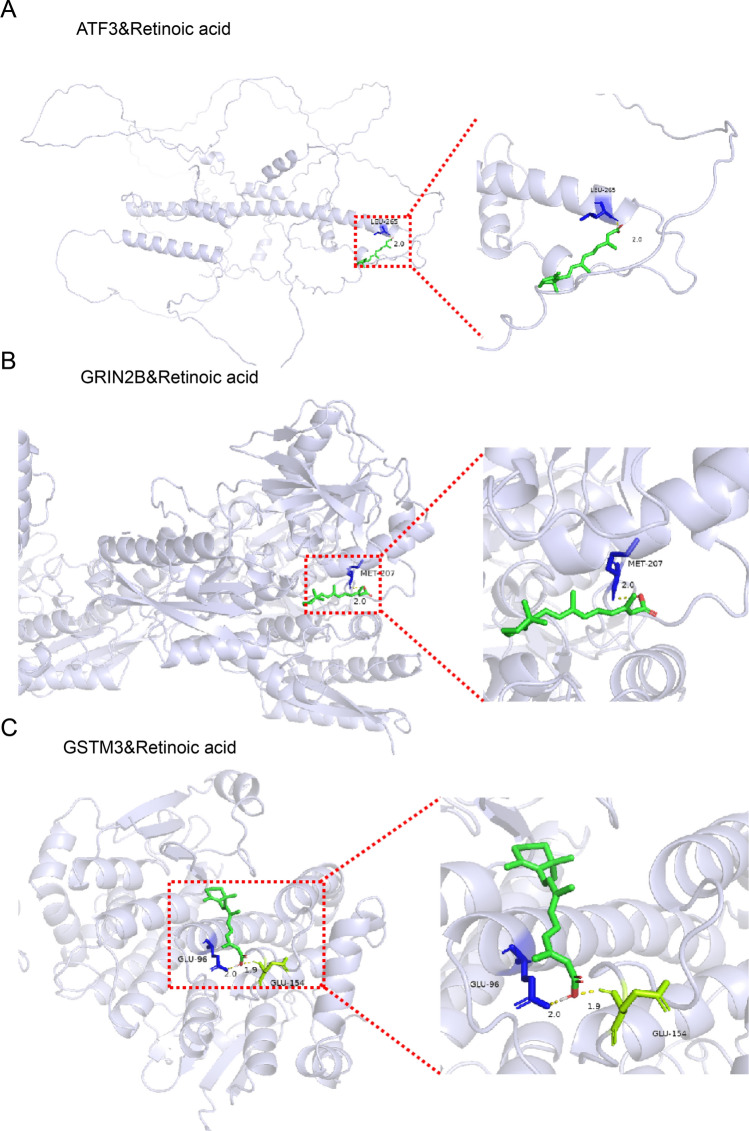
Expression levels of these biomarkers were examined in corneal stromal tissues obtained from patients undergoing SMILE surgery. (**A**) Quantitative reverse transcription PCR (RT-qPCR) (*n* = 6 per group) for mRNA expression levels of three biomarkers. (**B**) Western blot (*n* = 4 per group) for protein expression levels of three biomarkers. NS, not significant. *P < 0.05, **P < 0.01, ***P < 0.001. Low myopia: ≥ − 3.00 D and < 0 D); high myopia: ≤ − 6.00 D).

The original Article has been corrected.

