# Supplementary figures and images for: Gene expression and machine learning techniques uncover corneal biomarkers associated with oxidative stress in the myopia progression
Source: Sci Rep. 2026 Mar 30;16:10651. doi: 10.1038/s41598-026-46896-x (PMC13039845; doi:10.1038/s41598-026-46896-x)

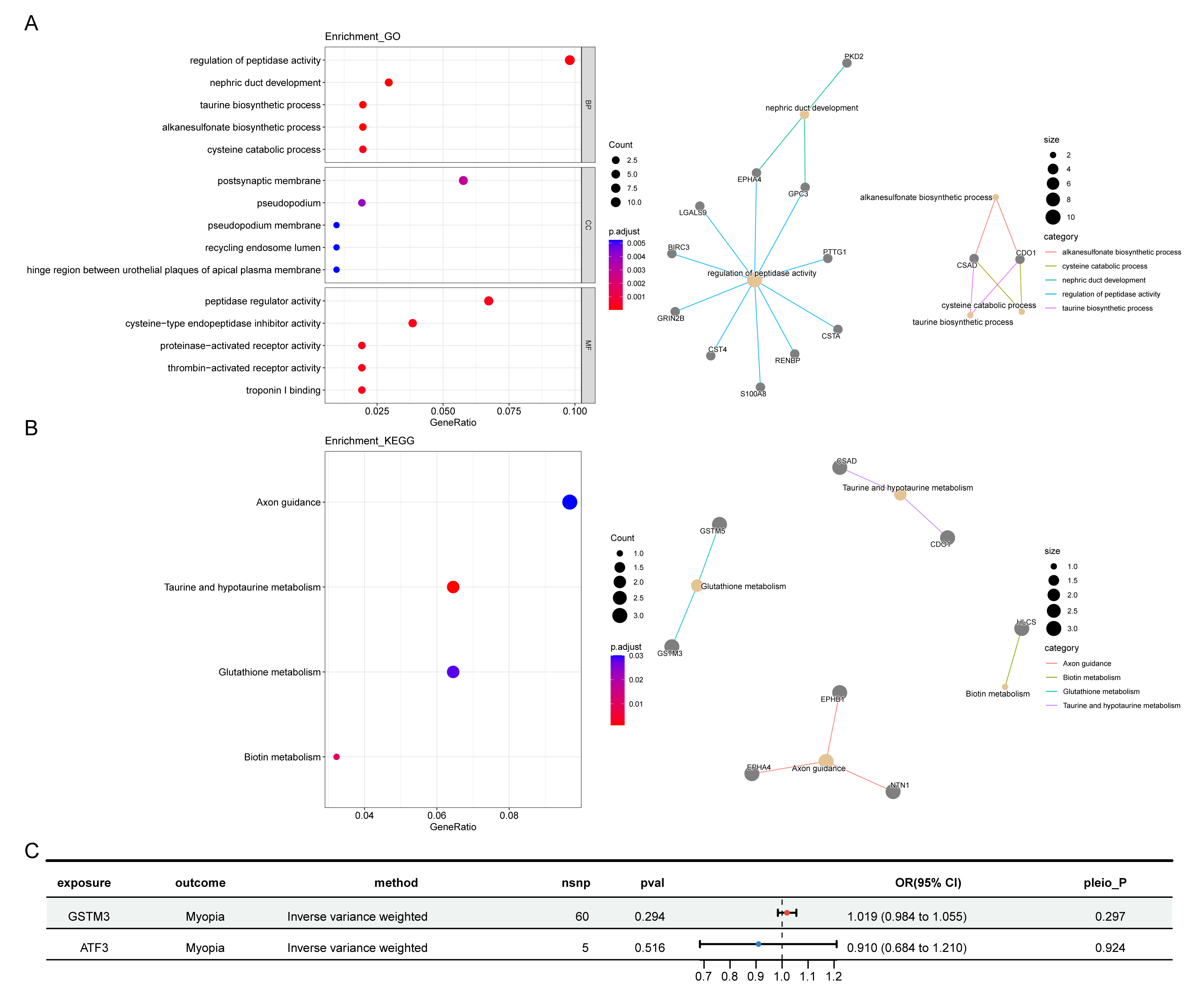

Supplement: Supplementary file 1 — Supplementary Material 1 [file 41598_2026_46896_MOESM1_ESM.tif]

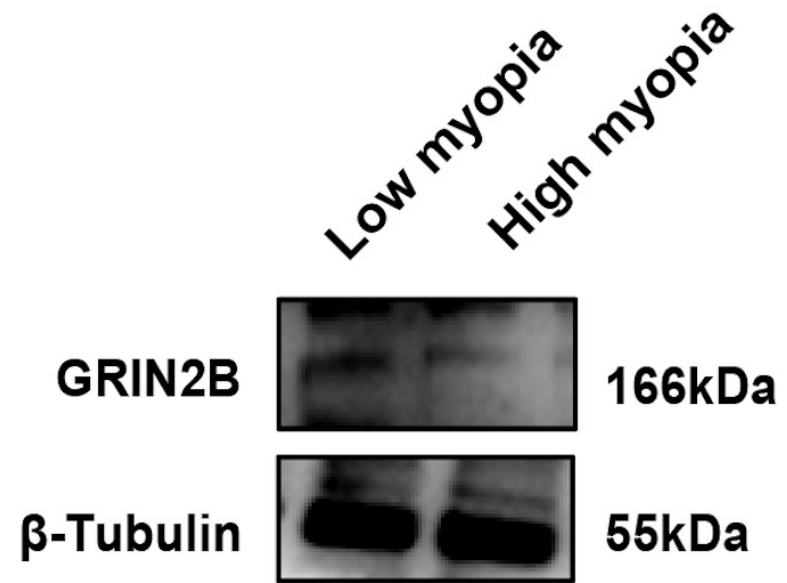

270KDA

165KDA

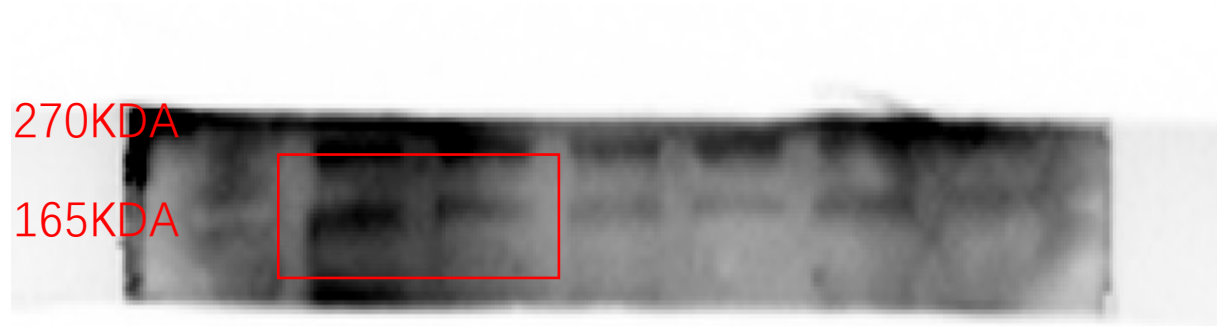

66KDA

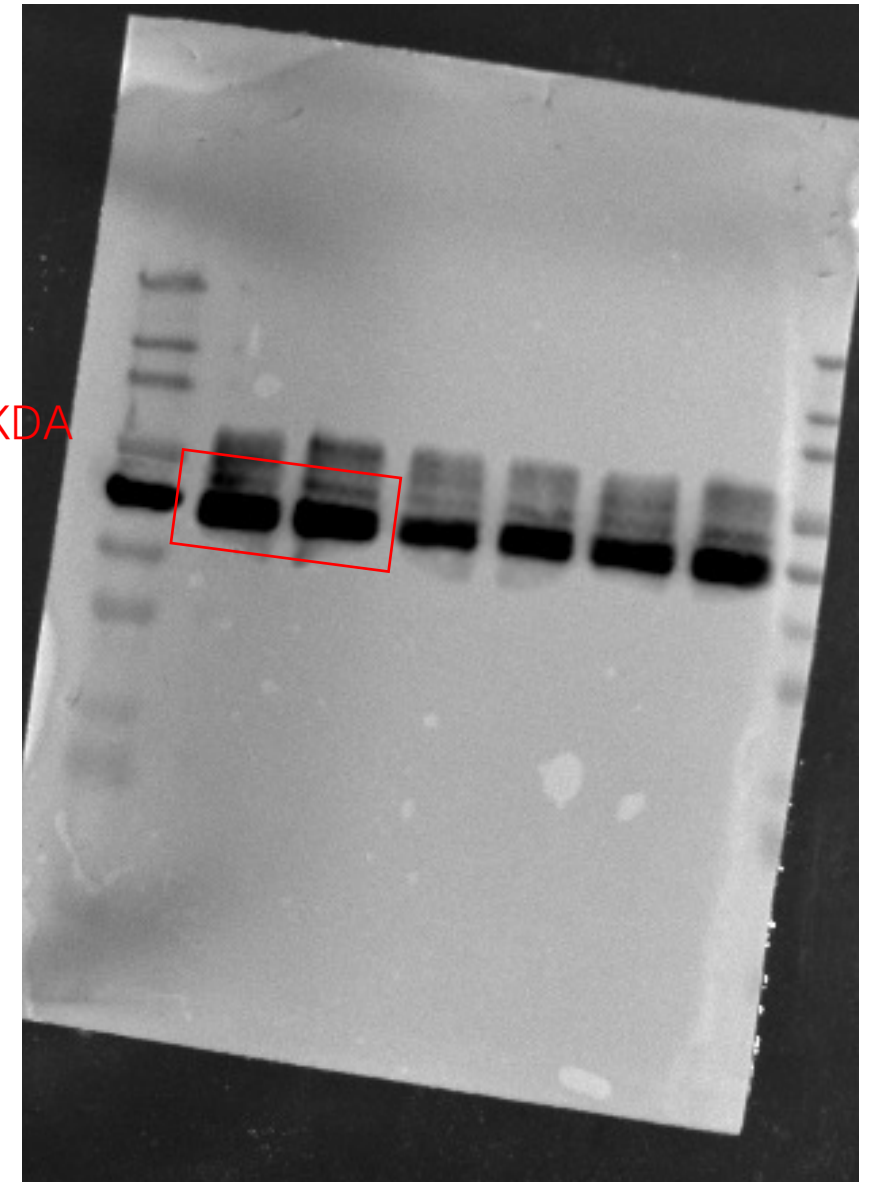

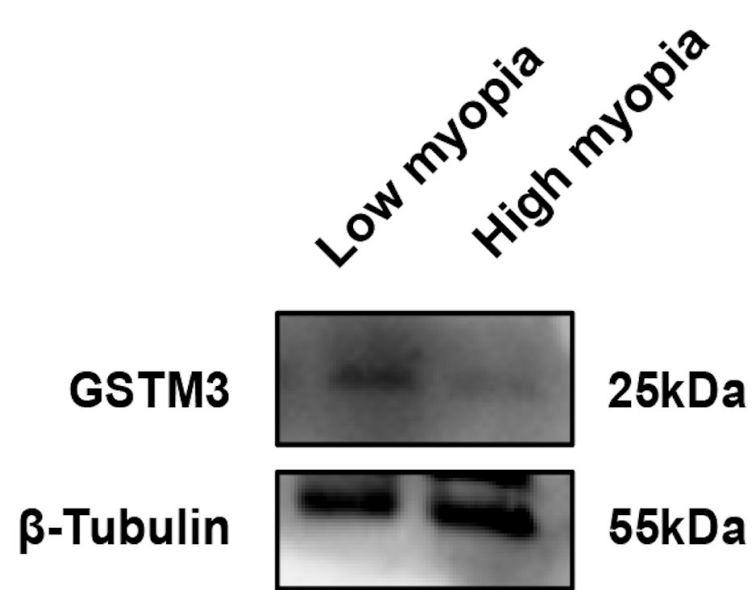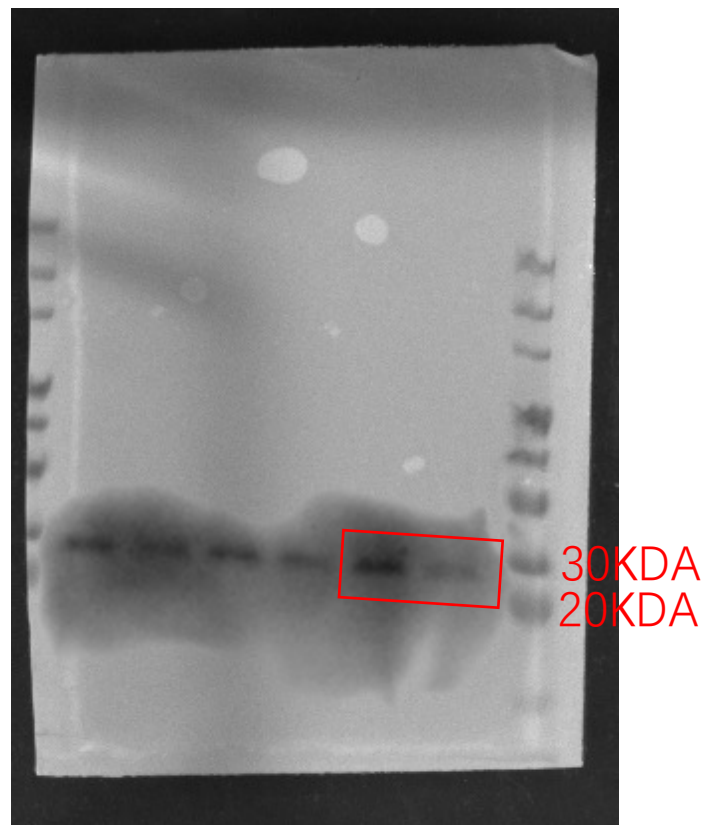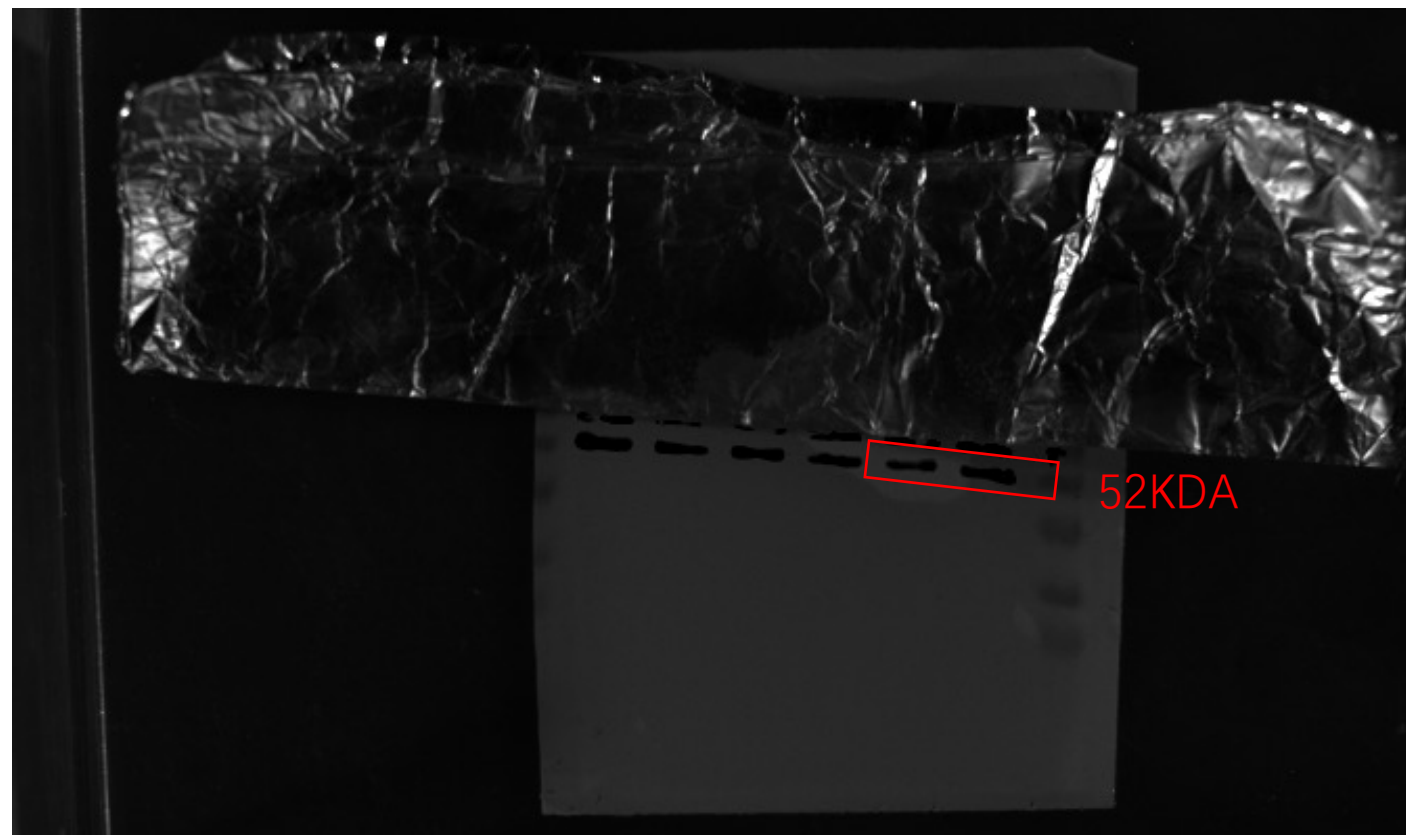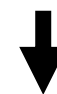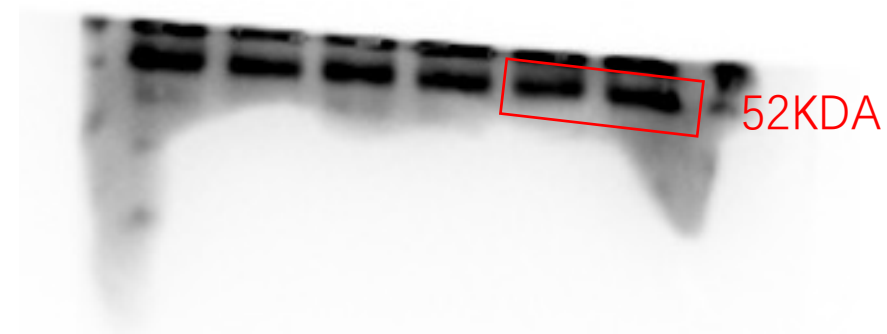

Low myopia  
High myopia

ATF3

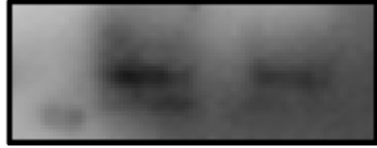

23/24kDa

$\beta$ -Tubulin

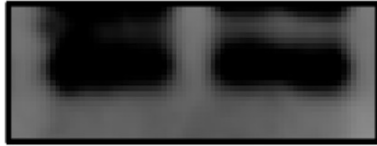

55kDa

20kDa

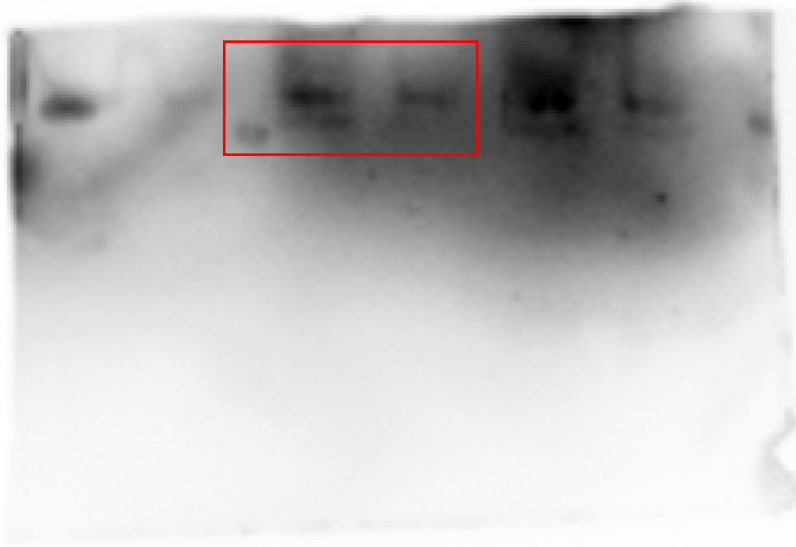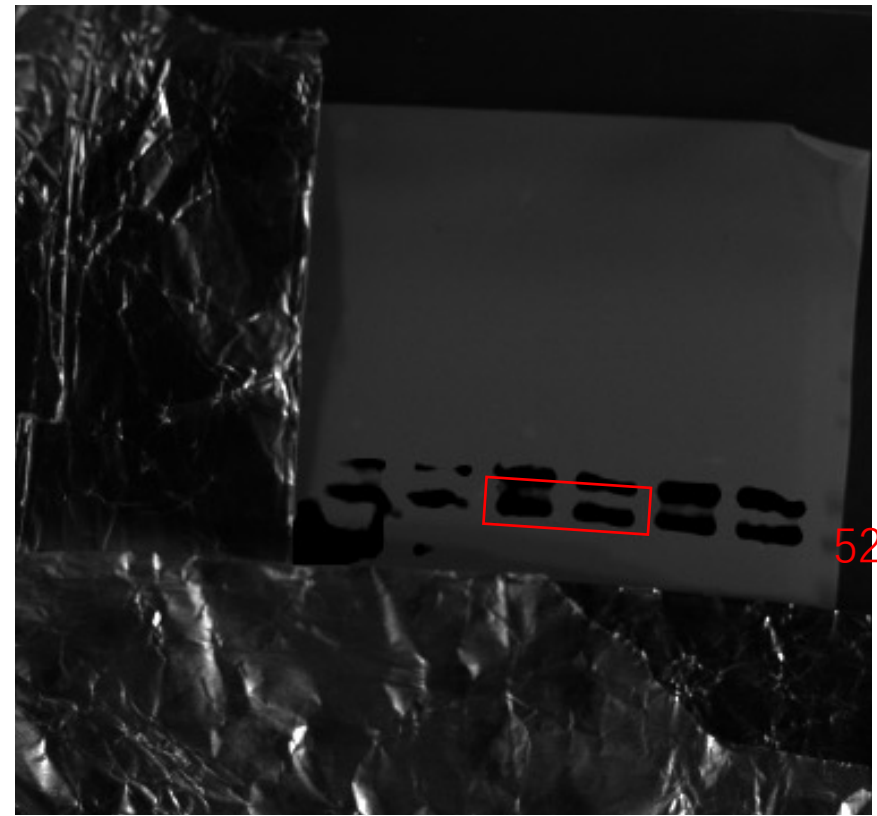

52kDa

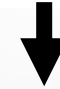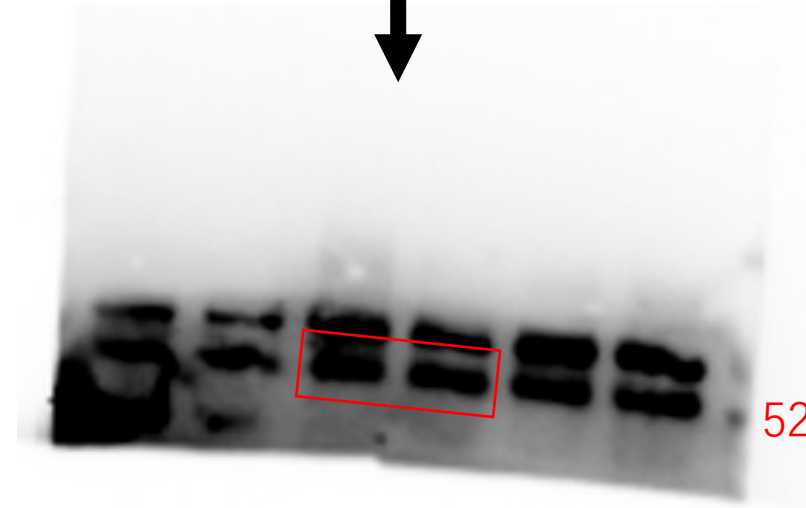

52kDa

Supplement: Supplementary file 7 — Supplementary Material 7 [file 41598_2026_46896_MOESM7_ESM.pdf]
