# Supplementary material for: Gene expression and machine learning techniques uncover corneal biomarkers associated with oxidative stress in the myopia progression
Source: Sci Rep. 2026 Mar 30;16:10651. doi: 10.1038/s41598-026-46896-x (PMC13039845; doi:10.1038/s41598-026-46896-x)
Supplement: Supplementary file 2 — Supplementary Material 2 [file 41598_2026_46896_MOESM2_ESM.docx]

**Supplementary Table 1 Primer sequences for biomarkers.**

| Gene | Sequence 5'-3' |
| --- | --- |
| GSTM3-F | GCGGACTGACTCACTCCATC |
| GSTM3-R | CCCCATGACATATCTCTTCTCCT |
| GRIN2B-F | CAGCAAAGCTCGTTCCCAAAA |
| GRIN2B-R | GTCAGTCTCGTTCATGGCTAC |
| ATF3-F | TTTGCTAACCTGACACCCTTTG |
| ATF3-R | AGAGGACATCCGATGGCAGA |
| GAPDH-F | AGGTCGGTGTGAACGGATTTG |
| GAPDH-R | GGGGTCGTTGATGGCAACA |
