# Supplementary material for: Gene expression and machine learning techniques uncover corneal biomarkers associated with oxidative stress in the myopia progression
Source: Sci Rep. 2026 Mar 30;16:10651. doi: 10.1038/s41598-026-46896-x (PMC13039845; doi:10.1038/s41598-026-46896-x)
Supplement: Supplementary file 6 — Supplementary Material 6 [file 41598_2026_46896_MOESM6_ESM.docx]

**Figure legend**

**Figure S1** (A-B) Gene Ontology (GO) and Kyoto Encyclopedia of Genes and Genomes (KEGG) pathway analyses for DEGs. (C) Forest plots for Mendelian randomisation (MR) analysis of biomarkers and myopia.
