# Supplementary material for: Gene expression and machine learning techniques uncover corneal biomarkers associated with oxidative stress in the myopia progression
Source: Sci Rep. 2026 Mar 30;16:10651. doi: 10.1038/s41598-026-46896-x (PMC13039845; doi:10.1038/s41598-026-46896-x)
Supplement: Supplementary file 8 — Supplementary Material 8 [file 41598_2026_46896_MOESM8_ESM.docx]

Raw western-blot image


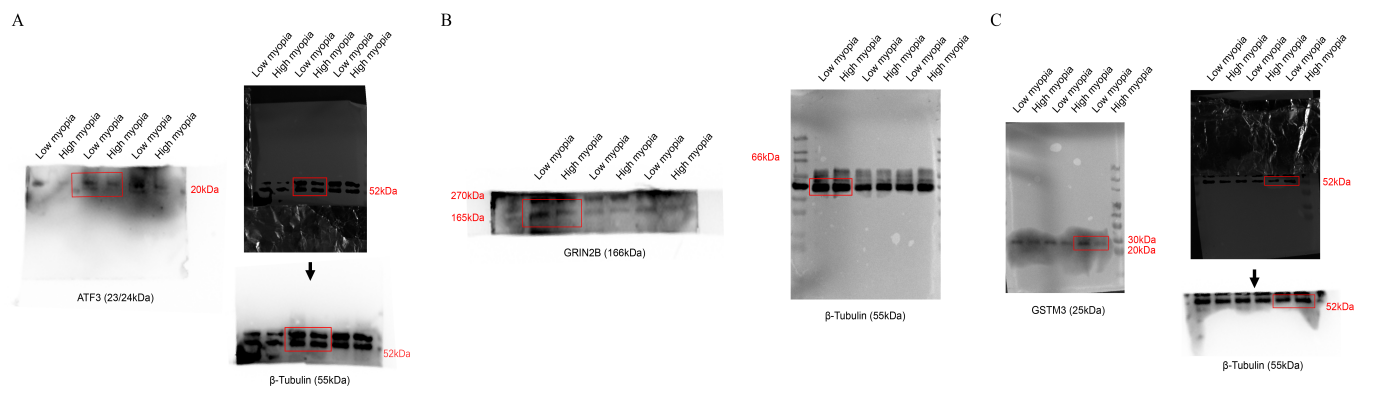


**Figure 1. Original images for Western blotting in figure 8. A:** Western blotting for ATF3 (23/24kDa) and β-Tubulin (55kDa), **B**: Western blotting for GRIN2B (166kDa) and β-Tubulin (55kDa), **C:** Western blotting for GSTM3 (25kDa) and β-Tubulin (55kDa).
